# Stable Isotope Fractionation of Metals and Metalloids in Plants: A Review

**DOI:** 10.3389/fpls.2022.840941

**Published:** 2022-04-19

**Authors:** Matthias Wiggenhauser, Rebekah E. T. Moore, Peng Wang, Gerd Patrick Bienert, Kristian Holst Laursen, Simon Blotevogel

**Affiliations:** ^1^Group of Plant Nutrition, Department of Environmental System Science, Institute of Agricultural Sciences, ETH Zurich, Zurich, Switzerland; ^2^MAGIC Group, Department of Earth Science and Engineering, Imperial College London, London, United Kingdom; ^3^College of Resources and Environmental Sciences, Nanjing Agricultural University, Nanjing, China; ^4^Crop Physiology, Molecular Life Sciences, Technical University of Munich, Freising, Germany; ^5^Plant Nutrients and Food Quality Research Group, Plant and Soil Science Section and Copenhagen Plant Science Centre, Department of Plant and Environmental Sciences, Faculty of Science, University of Copenhagen, Copenhagen, Denmark; ^6^Laboratoire Matériaux et Durabilité des Constructions (LMDC), UPS/INSA, Université Paul Sabatier - Toulouse III, Toulouse, France

**Keywords:** stable isotopes, fractionation, plant uptake, translocation, process tracing, multiple-collector-ICP-MS, metals, metalloids

## Abstract

This work critically reviews stable isotope fractionation of essential (B, Mg, K, Ca, Fe, Ni, Cu, Zn, Mo), beneficial (Si), and non-essential (Cd, Tl) metals and metalloids in plants. The review (i) provides basic principles and methodologies for non-traditional isotope analyses, (ii) compiles isotope fractionation for uptake and translocation for each element and connects them to physiological processes, and (iii) interlinks knowledge from different elements to identify common and contrasting drivers of isotope fractionation. Different biological and physico-chemical processes drive isotope fractionation in plants. During uptake, Ca and Mg fractionate through root apoplast adsorption, Si through diffusion during membrane passage, Fe and Cu through reduction prior to membrane transport in strategy I plants, and Zn, Cu, and Cd through membrane transport. During translocation and utilization, isotopes fractionate through precipitation into insoluble forms, such as phytoliths (Si) or oxalate (Ca), structural binding to cell walls (Ca), and membrane transport and binding to soluble organic ligands (Zn, Cd). These processes can lead to similar (Cu, Fe) and opposing (Ca vs. Mg, Zn vs. Cd) isotope fractionation patterns of chemically similar elements in plants. Isotope fractionation in plants is influenced by biotic factors, such as phenological stages and plant genetics, as well as abiotic factors. Different nutrient supply induced shifts in isotope fractionation patterns for Mg, Cu, and Zn, suggesting that isotope process tracing can be used as a tool to detect and quantify different uptake pathways in response to abiotic stresses. However, the interpretation of isotope fractionation in plants is challenging because many isotope fractionation factors associated with specific processes are unknown and experiments are often exploratory. To overcome these limitations, fundamental geochemical research should expand the database of isotope fractionation factors and disentangle kinetic and equilibrium fractionation. In addition, plant growth studies should further shift toward hypothesis-driven experiments, for example, by integrating contrasting nutrient supplies, using established model plants, genetic approaches, and by combining isotope analyses with complementary speciation techniques. To fully exploit the potential of isotope process tracing in plants, the interdisciplinary expertise of plant and isotope geochemical scientists is required.

## Introduction

Stable isotopes are atoms with the same number of protons and electrons but a different number of neutrons and are considered stable if they do not undergo a measurable nuclear decay. Isotope fractionation describes small changes in the isotope composition (i.e., relative isotope abundances) during chemical reactions or physical processes. Isotope compositions of plant organs can provide a record of biological and physico-chemical processes that control an element’s journey from their source to different organs, such as roots, leaves, and fruits. This information is complementary to organ-specific concentrations that provide information on element availability and utilization. This complementary information has been widely used for so-called “traditional isotopes” of, for example, H, C, N, O, and S to investigate for example water and fertilizer utilization, as well as nutrient assimilation (O’Leary et al., 1992; Tcherkez and Tea, 2013; Novak et al., 2019, 2021). Analytical advances during the past two decades now also allow the detection of naturally occurring variations of isotope compositions in plants for other, usually heavier elements (Hoefs, 2018), known as “non-traditional isotopes.” Here, we critically review the fractionation of non-traditional stable isotopes to identify biological and physico-chemical processes that control the uptake and transport of these elements within plants (i.e., stable isotope process tracing). To this end, we: (i) provide interdisciplinary fundamentals on plant physiology and stable isotope process tracing, (ii) compile available data on isotope process tracing of B, Mg, Si, K, Ca, Fe, Ni, Cu, Zn, Mo, Cd, and Tl, (iii) interlink and discuss findings and approaches from the individual element reviews, and (iv) highlight best practices for data acquisition and interpretation, and define future research priorities. This review does not cover the use of non-traditional stable isotopes to trace the origin of elements (i.e., “source tracing”) and to decipher complexation processes of elements onto soil components and thereby isotope fractionation between soil pools. Both topics have been reviewed recently (Komárek et al., 2021; Wang et al., 2021). Also beyond the scope of this review is the use of enriched stable isotopes as tracers to, for example, identify pathways of elements in plants (e.g., Yan et al., 2018). Finally, this review contributes to bridge plant science and isotope geochemistry by harmonizing nomenclature and working models of plant-based isotope studies to encourage interdisciplinary research and collaboration.

### Non-traditional Isotopes: The Basics

In plants, isotope fractionation is usually mass-dependent (Table 1). Mass-dependent isotope effects are induced by slight differences of masses of distinct isotopes. Properties affected by the mass difference are velocities and diffusivities, as well as vibrational and rotational frequencies and thermodynamic energies (Dauphas and Schauble, 2016). Differences in vibrational frequencies are the most important factor, as they determine isotope fractionation during chemical reactions. The general theory of isotope fractionation has been extensively reviewed elsewhere (Fry, 2006; Hoefs, 2018). We use Ca as an example in the following paragraphs to illustrate the basics of non-traditional isotope fractionation in plants. Isotope compositions are governed by equilibrium and kinetic isotope fractionation (Table 1). Equilibrium fractionation can occur when, for example, Ca ions in the soil or nutrient solution sorb to the negatively charged root apoplast (Cobert et al., 2011). In this case, thermodynamic equilibrium is reached when the adsorption and desorption of Ca ions are equally likely and occur at the same rate. Importantly, at isotope equilibrium, the individual Ca isotope ratios (e.g., ^44^Ca/^40^Ca) of the adsorbed and desorbed (i.e., dissolved) phase remain the same over time. At equilibrium conditions, the isotope fractionation depends on the mass difference of the isotopes and the stiffness of the bonds involved. Heavier isotopes preferentially go into stiffer bonds (higher spring constant of the bond, Bigeleisen, 1965). This effect is due to the larger mass-dependent difference in zero-point energy in stiffer bonds. In applied sciences, the stiffness of a bond is often approximated by the bond length or the thermodynamic stability of a chemical species.

**Table 1:** Glossary of terms relevant to isotope composition analysis.

Analytical precision	Usually expressed as the 2× standard deviation (2sd) of repeat measurements of a primary/bracketing standard or matrix-matched reference material (Supplementary Tables S1, S2), or the 2sd of repeated sample measurements. Gives a good estimate of the isotope composition resolvability. Often referred to as “external precision.”
Analyte purification	Isolation of analyte from the matrix of a digested sample. Most common method is ion exchange chromatography: percolation of acids of different molarities through resin-filled columns.
Digestion	Breakdown of molecules to allow liberation of individual elements. Uses hotplate or microwave oven-assisted acid digestion using nitric acid (and hydrogen peroxide) /aqua-regia /perchloric acid /hydrofluoric acid. For Si analysis, alkaline fusion (e.g., using NaOH) is more appropriate, since SIF_6_ is volatile after HF digestion.
Double spike	A mass bias correction technique for elements with ≥ 4 stable isotopes. A mixture of 2 enriched isotope solutions with known concentration and isotope composition is equilibrated with samples. An iterative procedure is used to deconvolute the data.
Equilibrium fractionation	Occurs when two or more substrates are in chemical (isotope) equilibrium. Heavier isotopes are preferentially incorporated into stiffer bonds in equilibrium reactions.
External normalization	A mass bias correction technique using a second element that is close in atomic mass and ionization potential to the analyte.
Isobaric interference	When isotopes of different elements have similar mass, mass spectrometry may be unable to distinguish between them, for example, ^40^Ca, ^40^Ar.
Isotope fractionation	Changes in proportions of isotopes that occur during chemical reactions or physical processes.
Kinetic (isotope) fractionation	In chemical reactions that are not at equilibrium, light isotopes accumulate in reaction products as they have slightly lower activation energies and therefore react faster.
Mass bias	Isotope fractionation that occurs inside mass spectrometers due to differences in transmission efficiencies between isotopes. This can induce large isotope fractionations, so post-measurement mathematical correction is required. Mass bias is much stronger in ICP than in TIMS.
Mass-dependent fractionation (MDF)	Fractionation scales with the difference in isotopic mass. for example, the mass difference between ^68^Zn and ^64^Zn is ~double that of ^66^Zn and ^64^Zn, so fractionations of ~2 and ~1‰ are expected (Maréchal et al., 1999).
Molecular interference	Also known as poly-atomic interferences. These occur within mass spectrometers when the mass of a molecule (e.g., ^40^Ar^16^O) overlaps with an isotope of the analyte (e.g., ^56^Fe).
Multiple collector inductively coupled plasma mass spectrometry	Use an Ar ICP source, magnetic sector analyzer, and Faraday collector array to simultaneously measure isotope abundances at precisions higher than attainable by single-collector ICP-MS. Enables analysis of a large variety of elements, including those with high ionization potentials, such as Zn. Samples are introduced as a solution or aerosol and even solid when laser ablation is used.
Procedural blank	A sample taken through every preparation step to enable the quantification of any contamination that can alter a samples’ true isotope composition.
Sample-standard bracketing	A mass bias correction technique. Samples are analyzed between standards. Post-measurement, a correction is made relative to the standard before and after the sample.
Thermal ionization mass spectrometry (TIMS)	More traditional devices for isotope analysis. Samples are deposited on a filament that is heated by an electric current to ionize the analyte. Isotopes are separated using a magnetic sector and an array of faraday cups. TIMS measurements involve lower mass bias and interferences than MC-ICP-MS, however they are slower and accurate mass bias correction mostly relies on the double spike technique, drastically limiting the amount of possible analytes.

If a system is not in thermodynamic equilibrium, kinetic processes control the isotope fractionation. For example, during the precipitation of Ca oxalate in plants (Cobert et al., 2011). Precipitation can induce kinetic isotope fractionation as long as the quantity of mineral Ca oxalate is increasing. At this stage, the forward reaction is quicker than the backward reaction (i.e., the dissolution of Ca oxalate) and favorably light isotopes precipitate into Ca oxalate. This is due to the slightly lower activation energy of lighter isotopes. When the amount of Ca oxalate remains constant, an (isotope) equilibrium between mineral and solution will be reached and the resulting isotope fractionation is governed by equilibrium fractionation. Note that chemical equilibrium is usually established faster than isotope equilibrium.

Isotope fractionation differs in closed and open systems, which can be defined using Rayleigh modeling (Fry, 2006). In closed systems, only negligible quantities of reactants or reaction products are added or removed from a system. For instance, a plant that preferentially takes up light Ca isotopes from a nutrient solution will deplete the nutrient solution of Ca and light Ca isotopes if the nutrient solution is not frequently replenished. As the plant will continue to preferentially remove light isotopes from the nutrient solution, the nutrient solution will become enriched with heavy isotopes while the isotopes taken up by the plant become successively heavier. Finally, when the entire Ca is taken up from the solution, the plant will have the same Ca isotope ratio as the solution initially had. In contrast, if the solution is frequently replenished, the plant is virtually exposed to an infinite Ca source and the system is considered open. In such a system, the plant will continuously take up light Ca isotopes from the nutrient solution and the Ca isotope ratios in the plant and in the solution do not change over time.

Isotope ratio analyses are more meaningful when mass balances are calculated. To calculate such “isotope mass balances” in plants, the dry weights, the plant organs (e.g., roots, leaves) concentrations, and isotope ratios of both the plant organs and the nutrient source need to be determined. For instance, the weighted mean of Ca isotope ratios in different plant organs provide the isotope ratio of the whole plant and thereby the isotope fractionation associated with Ca uptake from soil to plant can be calculated. Note, these calculations consider that there is no significant efflux of Ca from the plant into the soil.

### Metals and Metalloids in Plants: The Basics

Plants require 17 elements in order to complete their life cycle (Table 2). Besides hydrogen (H), carbon (C), and oxygen (O), which are the main building blocks of plant tissues, the remaining 14 elements are the macronutrients: nitrogen (N), magnesium (Mg), phosphorus (P), sulfur (S), potassium (K), calcium (Ca) and the micronutrients: boron (B), chlorine (Cl), manganese (Mn), iron (Fe), nickel (Ni), copper (Cu), zinc (Zn) and molybdenum (Mo) (Marschner and Marschner, 2012).

**Table 2:** Overview of elements found in plant tissue that have been studied using stable isotope fractionation (listed according to atomic weight)^**^.

Element	Symbol (atomic weight)	Main plant available form(s)	Normal/sufficient content (in dry matter*)	Phloem mobility	Functional roles in plants
Boron	B (10.81)	B(OH)_4_^−^ B(OH)_3_	5–60 μg/g	Low	***Essential*** plant nutrient: Stabilization of cell walls, nucleotide biosynthesis, translocation, pollen germination
Magnesium	Mg (24.305)	Mg^2+^	0.1–1%	High	***Essential*** plant nutrient: Photosynthesis (central atom of chlorophyll), enzyme activation
Silicon	Si (28.085)	H_4_SiO_4_	0.1–10%	Low	***Beneficial*** plant nutrient: Alleviates abiotic and biotic stresses (salt, heavy metal, fungal disease, osmotic, drought)
Potassium	K (39.098)	K^+^	1–6%	High	***Essential*** plant nutrient: Translocation, osmoregulation, pH homeostasis, enzyme activation
Calcium	Ca (40.078)	Ca^2+^	0.2–3%	Low	***Essential*** plant nutrient: Cell wall synthesis and stabilization, signal transduction
Iron	Fe (55.845)	Fe^3+^ Fe^2+^	30–300 μg/g	Conditional	***Essential*** plant nutrient: Enzyme activation, electron transport, chlorophyll biosynthesis, oxidative stress protection
Nickel	Ni (58.693)	Ni^2+^	0.1–1 μg/g	Conditional	***Essential*** plant nutrient: Enzyme activation, urea metabolism
Copper	Cu (63.546)	Cu^+^ Cu^2+^	4–25 μg/g	Conditional	***Essential*** plant nutrient: Enzyme activation, synthesis of cell wall components, electron transport, oxidative stress protection
Zinc	Zn (65.38)	Zn^2+^	15–80 μg/g	Conditional	***Essential*** plant nutrient: Gene expression, enzyme activation, hormone and nucleic acid synthesis, oxidative stress protection
Molybdenum	Mo (95.95)	MoO_4_^2−^	0.1–1 μg/g	Conditional	***Essential*** plant nutrient: Enzyme activation, nitrogen fixation, nitrate reduction
Cadmium	Cd (112.41)	Cd^2+^	0.01–1 μg/g	Conditional	***Non-essential, non-beneficial***
Thallium	Tl (204.38)	Tl^+^	site dependent	NA	***Non-essential, non-beneficial***

Main plant available form(s) of each element, normal or sufficient content, phloem mobility, functional roles in plants and selected additional information of relevance for interpretation of element-specific isotope fractionations. NA = information not available.

* Dependent on the developmental stage of the plant, the plant species, the plant genotype, and the plant organ.

** Adapted from Pilon-Smits et al. (2009); Husted et al. (2011); Marschner and Marschner (2012); de Bang et al. (2020); www.iupac.org. For element-specific information on uptake, functional roles and utilization in plants, we refer to recent reviews for: B (Yoshinari and Takano, 2017), Ca (Thor, 2019), Mg (Chen et al., 2018), K (Wang and Wu, 2017), Si (Ma, 2015), Cu, Fe, Ni, Zn, and Cd (Andresen et al., 2018), Mo (Tejada-Jimenez et al., 2018).

Besides the essential plant nutrients, many other elements of the periodic table are also found in plants. Some elements, such as silicon (Si), are considered beneficial for certain plant species. Others with no functional role are present in plant organs as a result of their environmental abundance and due to non-specific uptake by plants. Some of these elements represent a threat to plant growth and performance, for example, cadmium (Cd). Most elements are acquired as inorganic ions from the soil solution and are subsequently translocated in plant organs and utilized in a plethora of biochemical functions (Table 2).

#### Physiological Processes and Isotope Compositions in Plants

**Figure 1:**
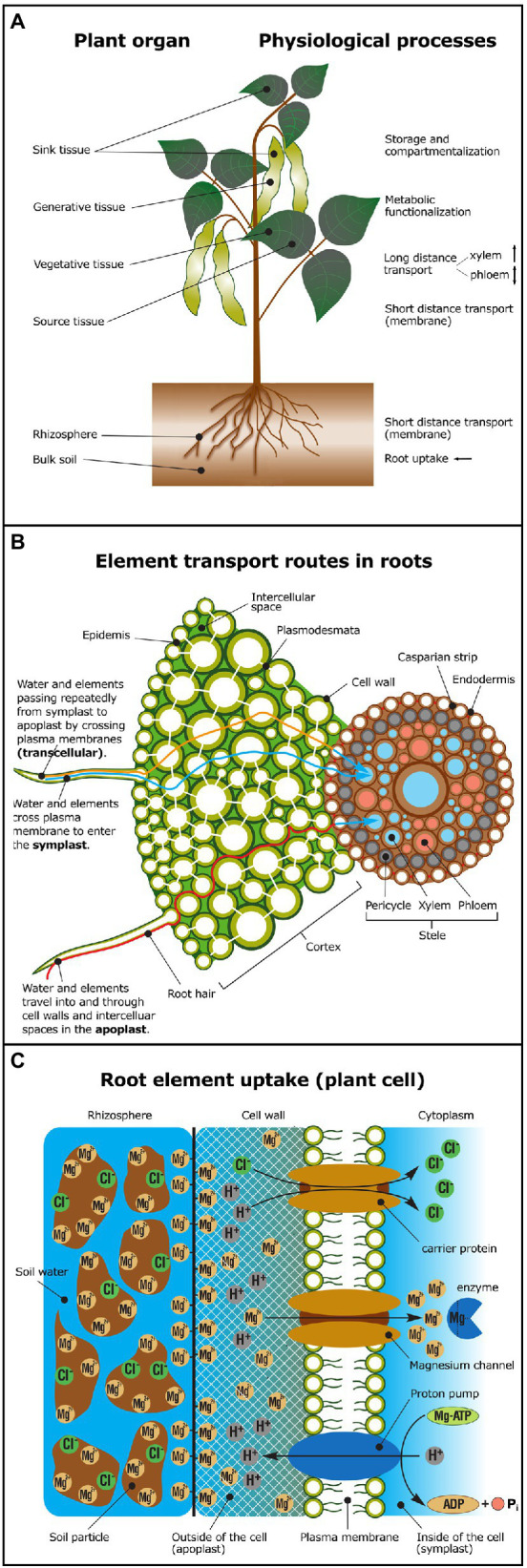
Overview of physiological processes potentially causing isotope fractionation. **(A)** In shoots, isotope fractionation may occur during short distance transport across membranes, during long-distance transport in xylem (unidirectional transport) or in the phloem (bidirectional transport), due to metabolic functionalization (e.g., enzyme activation, ligand exchange), element storage or compartmentalization. Major physiological processes in roots are nutrient uptake and short distance transport across membranes. Elements are taken up from the rhizosphere and translocated *via* the xylem to sink tissue (young leaves or generative tissue, such as fruits, grains, or pods). Elements may also be re-translocated *via* the phloem from source tissue (e.g., old leaves) to sink tissue. **(B)** Simplified model of symplastic (blue), apoplastic (red) versus transcellular (orange) element transport routes in plant roots. Cell-to-cell transport in the symplastic pathway occurs *via* plasmodesmata. In the apoplastic pathway elements travel in the cell wall and the intercellular space. The plasma membrane must be crossed to exit the root cortex and enter the stele and the vasculature system. **(C)** Elements may be bound to soil particles in the rhizosphere or to the negatively charged cell wall prior to uptake across the plasma membrane mediated by transport proteins (e.g., *via* carriers and channels). In the cytoplasm, elements may form complexes with enzymes and substrates—for example, ATP activation through Mg-ATP complexation (Adapted from Marschner and Marschner, 2012; Taiz et al., 2015). For glossary of terms and processes see Table 3.

Physiological processes, such as root uptake, short and long-distance transport (in xylem or phloem), storage (e.g., vacuolar), metabolic functionalization, and compartmentalization (Figure 1A), are a series of physico-chemical processes that potentially change isotope compositions of a given element. Physico-chemical processes comprise water mass flow, sorption, ligand exchange, redox changes, precipitation, dissolution, and diffusion.

#### Plant Root Uptake of Elements

Plants take up most elements in specific, phytoavailable, chemical forms from the soil solution (Tables 2, 3). These are transported in roots *via* the apoplastic, symplastic, or transcellular pathways (Figure 1B; Table 3). Elements enter the root apoplast with water mass flow, mainly driven by the transpiration stream or by diffusion along an electrochemical gradient. The apoplastic mobility of elements is determined by the number and affinity of available adsorption, complexation, and exchange sites within cell walls for the specific element (Meychik et al., 2021). When a physico-chemical barrier restricts further apoplastic transport, elements have to cross a cell membrane to take the symplastic and transcellular pathways, which may require metabolic energy (Figure 1C; Table 3). One such barrier during root-to-shoot transport is the endodermis. It forms the boundary between the cortex and the stele and contains a lignin- and suberin-based hydrophobic radial cell wall “impregnation” called the “Casparian strip” (Geldner, 2013). To cross the endodermis, all elements have to enter the intracellular space at least temporarily (Figure 1B). After crossing the endodermis, an element is loaded into the vascular system (Table 3) for further root-to-shoot transport.

**Table 3:** Glossary of terms and processes relevant for understanding isotope fractionation in plants (in alphabetical order).

Active transport cellular influx and efflux short distance transport	Movement of an ion/molecule across the membrane against the concentration gradient of the transported atom/molecule and/or against the electrochemical gradient. Requires metabolic energy to pump molecules across a membrane (transport direction = uniport; transport rates = 1–10^2^ molecules per sec; energization = ATP/PPi -hydrolysis; protein types mediating the active transport = pumps, ATPases, which demands structural changes of the protein; examples = Ca, Zn, Mn, Cd, Co P-type ATPases, Cd, Pb, As, Hg (II), Sb ATP-Binding-Cassette (ABC)-transporters).
Apoplastic transport (extracellular)	Transport of an ion/molecule in the apoplast, which comprises the entirety of plant cell wall and the intercellular space, without entering the cell. Note, elements that fulfill functions in the apoplast, like Ca (cell wall stabilization), can be considered as taken up by the plant although they have not entered the symplast.
Bulk Soil	Soil outside of the rhizosphere and neither penetrated nor influenced by plant roots.
Compartmentalization	Separation of ion/molecule into isolated subcellular compartments (organelles, cell wall, etc.) or cells.
Dual-affinity transport proteins	Transport proteins which are suggested to transport efficiently at high or low nutrient concentrations due to their ability to switch their structural or biochemical protein properties *via* post-translational modifications (e.g., the potassium transporter AtKUP1).
Electrochemical Gradient	A difference in 1) charge and 2) the chemical concentration of a compound across the plasma membrane.
High-affinity transport proteins	Transport proteins which are suggested to dominantly transport at low nutrient concentrations; transport can be saturated (e.g., the iron transporter AtIRT1).
Ligand	A group, ion or molecule coordinated to a central atom or molecule in a complex. For example nicotianamine, phytate, citrate and malate.
Long-distance transport (*via* mass flow)	Transport of ion/molecule within the vasculature at the level of the whole plant linking a multitude of roots with branches and leaves *via* the specialized tissues called xylem (unidirectional transport, up in a plant ↑) and phloem (bidirectional transport, up and down in a plant ↕). Driven by pressure flow (hydrostatic or osmotic).
Low-affinity transport proteins	Transport proteins which are suggested to dominantly transport at high nutrient concentrations; transport seems non-saturable (e.g., the cation Cd transporter OsLCT1).
Passive transport cellular influx and efflux short distance transport	Movement of an ion/molecule across the membrane along the concentration gradient of the transported atom/molecule and/or along the electrochemical gradient. Protein-mediated or *via* sole diffusion across the membrane (transport direction = uniport; transport rates = 10^6^–10^8^ molecules per sec; energization = electrochemical potential gradient; protein types mediating the passive transport = channels; examples = K, B, Si, Cl, Ca channels).
Phytoavailability	Plant availability - i.e., elements are available to plants for uptake. Most commonly used when referring to essential plant nutrients that are in the right chemical form at sufficient concentrations in the soil solution.
Rhizosphere	The soil around living roots, which is influenced by root activity. The radial extension of the rhizosphere can range from sub-μm to supra-cm scales.
Secondary active transport cellular influx and efflux short distance transport	Movement of an ion/molecule across the membrane against the concentration gradient of the transported molecule and/or against the electrochemical gradient (transport direction = uniport, symport, antiport; transport rates = 10^2^–10^4^ molecules per sec; energization = proton (H^+^) gradient established by active transport processes; protein types mediating the active transport = carriers, which demands structural changes of the protein; examples = K, Na, Fe, Zn, Mg, B, Cl transporters).
Selectivity of a transporter	Each individual transport protein, irrespective of whether it is a channel (passive transporter), a carrier (a secondary active transporter) or an active transporter (pump) has a specific transport selectivity allowing for selective passage of (a) specific atom(s)/molecule(s). This transporter-intrinsic specificity does neither depend on the type of transport process (active vs. passive) nor on the transported atom/molecule (ionic versus polar molecule).
Short distance transport	Transport that occurs within, into and out of cells including a membrane crossing in the process. Includes diffusion and mass flow.
Symplastic (intracellular) transport	Cell-to-cell transport of an ion/molecule *via* plasmodesmata which has once entered the symplast *via* passive diffusion or a transport protein.
Transcellular (intracellular/extracellular) transport	Transport protein-mediated transport of an ion/molecule from one cell to the other, passing repeatedly from symplast to apoplast, independent of the water mass flow but depending on a non-overlapping localization of influx and efflux transporters in the plasma membrane to allow a directional flow.

#### Cross-Membrane Transport of Elements

Elements are transported across membranes either by passive diffusion through the phospholipid bilayer or *via* an armada of specialized membrane transport proteins (Figure 1C; Table 3). Passive diffusion is negligible for charged molecules due to their restricted membrane permeability while passive channels, secondary active carriers, and active transporters generate an element-specific and rapidly controllable pathway through various membranes (Stein and Litman, 2015; Tang et al., 2020). Each transport protein has a unique activity. The subcellular localization of each membrane protein is mostly restricted to one cellular membrane system (e.g., plasma membrane, tonoplast, mitochondrial or chloroplastic membrane) or even a spatially targeted fraction of a membrane system. For instance, certain transporters that control the uptake of nutrients, such as B and Si, are polarly localized solely at the distal side of root rhizodermis cells, while transporters responsible for the further translocation of nutrients into the cortex are localized to the proximal side (Ma and Yamaji, 2015; Yoshinari and Takano, 2017). Similar to animal epithelial cells, where the mechanisms for polar localization of nutrient transporters at the apical or basolateral membrane were intensively studied (Mellman and Nelson, 2008), many plant nutrient transporters that are either responsible for rhizodermal uptake or xylem loading, have been demonstrated to be polarly localized (Łangowski et al., 2010 and references therein).

The main driving force for protein-mediated membrane transport is an electrochemical gradient (Table 3) which depends on differences in element concentrations and electrical potential inside and outside of the cell. The electrochemical gradient is established by membrane proteins (H^+^-ATPases), which pump protons out of cells against an inward directed proton gradient (Figure 1C). This process requires metabolic energy from ATP hydrolysis and generates an electrical gradient (positive outside, negative inside) and a pH gradient (acidic outside vs. neutral/alkaline inside) across the membrane. As a consequence, there is a permanent driving force (proton motive force) for cations into plant cells and anions out of plant cells (Falhof et al., 2016). These energetic downhill transport processes can be used to energize uphill transport processes that are mainly facilitated by carrier-type transport proteins (Stein and Litman, 2015; Tang et al., 2020).

Specific uptake and efflux transport proteins have been identified for mineral nutrients in the rhizodermis and root hairs at the plant–soil interface (Bienert et al., 2021), as well as in the cortex cells which have to be passed before nutrients enter the vasculature of the stele (Figure 1B). Although transport proteins have often developed a high selectivity for their substrate-of-interest, most are not fully element-specific and can accommodate a range of substrates simply due to physico-chemical similarities. The selectivity of transport proteins is mostly determined by 3D physico-electrochemical properties of the amino acid residues constituting the surface of and the transport pathway through the protein (Chan and Shukla, 2021). While some transporters are ubiquitously expressed, others are only found at certain developmental stages, times of day, or only in response to stimuli (e.g., nutrient deficiency, mineral toxicity).

In the absence of knowledge on transport proteins, nutrient uptake fluxes have been historically differentiated into low- and high-affinity uptake systems (Table 3). Low-affinity uptake systems act at higher phytoavailable nutrient concentrations while high-affinity uptake systems act at low phytoavailable nutrient concentrations. In addition, dual-affinity transport systems act under a wide concentration range of phytoavailable nutrient concentrations. The high-low-dual-affinity terminology is sometimes confusingly used as it is often extended to the binding affinity of a substrate to a transport protein that also determines nutrient uptake kinetics. However, underlying experimental transporter characteristics have not been determined and environmental parameters might determine transport rates by the respective protein (Dreyer and Michard, 2020).

#### Long-Distance Transport of Elements

From the root cortex, elements are transported into the stele and the vasculature system for long-distance, unidirectional transport toward the shoot (translocation, Figure 1A; Table 3). The xylem transports elements with the transpirational flow of water toward the leaves and generative tissues. Re-translocation of elements and bidirectional transport occurs in the phloem where high concentrations of assimilates (mainly sucrose) are loaded from source tissues (e.g., leaves) and are unloaded in sink tissues (e.g., fruits) by transport proteins. This generates a diffusion or osmotic gradient which induces a flow in the phloem and thereby long-distance transport of elements. The unloading of elements from the xylem and phloem as well as the distribution to physiological sink cells and organs again requires membrane transport proteins (see Cross-Membrane Transport of Elements). All these transport processes may cause isotope fractionation and change depending on physiological status, developmental stage, nutritional status, and the environmental condition at which a plant grows.

#### Binding of Elements to Organic Molecules

While elements are transported to their physiological destination they are exposed to a variety of non-transport related reactions. For instance, B readily forms ester bonds in many metabolites of primary and secondary metabolism while Cu can be retained in prosthetic bonds or as a cofactor in enzymes (Brdar-Jokanović, 2020; Li et al., 2020; Mir et al., 2021). Sorption, chelation, and complexation of elements to and with various organic and inorganic molecules is ubiquitous in plants. Some of these interactions are developmentally or physiologically targeted, others occur solely due to their chemical reactivity (Hanikenne et al., 2021). Noteworthy, in a quantitative sense, is the binding of, for example, Mg in chlorophyll, Fe in ferritin or heme, Zn in zinc finger proteins or nicotianamine, and trace metals to a plethora of thiols. In addition, the affinity of Ca to cell wall components contributes to the stability of cell walls (Thor, 2019).

## Methods for Isotope Analyses of Plants

Analyzing non-traditional stable isotope compositions of plants requires cautious sample handling, compared to traditional isotope analyses, and additional preparation steps, such as sample purification. In the following sections, the analytical procedures are listed chronologically.

### Sample Preparation

For all post-harvest processes, such as plant sample cleaning, drying, and homogenization, sample contamination should be kept to a minimum since it can significantly bias isotope compositions. For guidelines on post-harvest sample preparation, we recommend Hansen et al. (2013). A crucial step for isotope analyses in plants is the cleaining of the roots of hydroponically and soil grown plants. To remove soil particles, weak salt solutions, ultrapure, and ice-cold water (18.2 MΩ.cm) are used (Tang et al., 2016). Salt solutions or weak acids can be further used to extract the apoplastic root pool prior to isotope ratio analysis (e.g., Tang et al., 2016; Garnier et al., 2017). However, both the washing procedure and the extractant used may significantly impact the isotope ratios of the different root pools.

### Wet Chemistry

Wet chemistry sample preparation comprises two steps: sample digestion and analyte purification. To minimize sample contamination, these steps need to be conducted in “clean labs” (i.e., laboratories with non-metal fixtures, filtered air, ideally certified), ultra-clean reagents (e.g., distilled acids), and acid-cleaned PTFE/PFA containers (see also Section Quality Control). For most elements, digestion of plants requires strong acids (e.g., HNO_3_) and a microwave oven or a hotplate (Table 1). Silicon analyses represent a special case, where alkaline fusion procedures are more common than acid digestions (Frick et al., 2020). After digestion, the samples are “purified” by anion/cation exchange and extraction chromatography (Table 1; Supplementary Table S1) which is the most common technique to separate pure analyte fractions. Often more than one of these “column chemistry” procedures are required to separate residual matrix elements. It is important that the chromatographic yields are close to 100%, as any loss of element on the column can result in isotope fractionation and thereby bias results. For example, Cu isotopes can significantly fractionate between the first and last milliliter of elution from a commonly used column chemistry, and even not collecting the final 5% of Cu can induce a significant isotope fractionation (Maréchal and Albarède, 2002). For some elements, quantitative yields are less important if the double spike technique is used (Table 1; Supplementary Table S1).

### Isotope Analyses and Notation

To resolve differences in stable isotope composition in the per mil range, the use of multiple collector inductively coupled plasma mass spectrometry (MC-ICP-MS) or thermal ionization mass spectrometry (TIMS) is required (Table 1; Hoefs, 2018). Sample purification is necessary to avoid isobaric or poly-atomic/molecular interferences on the target element (Table 1). However, due to residual impurities or molecular interferences with elements present in the solution or the plasma (like N, O, H, Ar), it is often still challenging to perform precise isotope ratio analysis. Common problems include the formation of argides and those associated with analyzing isotopes that have very low natural abundances. For instance, ^56^Fe overlaps with the argide ^40^Ar^16^O. This problem is resolved by, for example, performing analyses at high-resolution modes, which is able to distinguish between Fe and argon oxides (Schoenberg and von Blanckenburg, 2005).

Mass bias during analyses needs to be corrected (Table 1). The simplest method adopted is “sample-standard bracketing” (Table 1). If a mass bias is non-linear, this method will not produce accurate results but can be improved by the addition of external normalization and mass fractionation laws (Table 1; Rehkämper et al., 2001). Such normalization, or “doping” uses the assumption that a dopant will undergo similar instrumental mass bias to the analyte, based on mass and ionization potential (e.g., Ni or Zn as doping element for Cu analyses). For elements with at least four stable isotopes, mass bias can be corrected by the double spike technique (Table 1; Rudge et al., 2009).

Stable isotope compositions are usually reported relative to an internationally recognized primary standard material and as a δ notation, exemplified for Ca (Hoefs, 2018):


(1) $$\delta^{44/40}Ca\;\left(‰\right)=\left[\frac{{\left({}^{44}Ca{/}^{40}Ca\right)}_{\mathrm{sample}}}{{\left({}^{44}Ca{/}^{40}Ca\right)}_{\mathrm{standard}}}-1\right]\;1000$$

Values are most often presented in per mil, but are also occasionally given as ε (10,000 times instead of 1,000 times multiplication). The isotopes used for reporting the isotope composition and the internationally accepted primary reference standard of each reviewed element are listed in Supplementary Table S1. For several elements, it is conventional that only the heavier isotope is included in the δ notation (e.g., δ^44/40^Ca is written as δ^44^Ca).

The isotope fractionation between two compartments of soil–plant system is expressed as:


(2) $$\Delta^{44}{Ca}_{a-b}\left(‰\right)=\delta^{44}{Ca}_{a}-\delta^{44}{Ca}_{b}$$

In plants, Δ can refer to the isotope fractionation (i) between the whole plant, a, and its source, b, resulting in Δ^44^Ca_plant-source_ to determine the isotope fractionation during the root uptake of an element or (ii) within a plant (e.g., Δ^44^Ca_shoot-root_). In isotope studies where full mass balances are difficult to calculate (e.g., for trees), often Δ^44^Ca_root-source_ is given. This notation is not equal to the isotope fractionation during uptake as the root isotope ratios result from both uptake and further transport of the element.

### Quality Control

To assure the quality of the non-traditional stable isotope ratio measurements, two principle quality control measures are necessary. First, the concentration of a “procedural blank” (Table 1) needs to be determined to ensure that the unintended addition of an analyte, for example, Zn, from acids, plastics, and dust, to a sample is kept to a minimum. As a general rule, a procedural blank that contains less than 1% of the analyte mass in a sample creates no significant artificial effect on isotope ratios. Second, reference samples need to be analyzed to show that the sample preparation and analyses are robust and reproducible. The quality of the isotope ratio measurement is commonly reported using the reproducibility of the primary reference standards (Supplementary Table S1). Furthermore, interlaboratory comparisons of certified reference materials (CRMs) enable the recovery of an element during sample digestion and purification to be calculated (Supplementary Table S2). There are published (although not certified) isotope ratios for some of these materials and we highly recommend their use, since they better match the composition of plant samples and enable the validation of the whole analytical process from sample preparation to isotope analyses.

## Isotope Fractionation In plants

In this section, isotope fractionation in plants is reviewed for individual elements. Where element mass balances were available mass balances were available, isotope fractionation upon uptake by plants (e.g., Δ^44^Ca_plant-source_) is reported, along with any within-plant isotope fractionation (e.g., Δ^44^Ca_shoot-root_, Δ^44^Ca_grains-leaves_). Interpretations on distinct isotope compositions of plants are reviewed when data-driven and well-substantiated hypotheses were provided. Elements that have been analyzed in exploratory studies, without detailed process tracing discussions (K, Mo, and Tl) are reviewed briefly at the end.

### Boron

**Figure 2:**
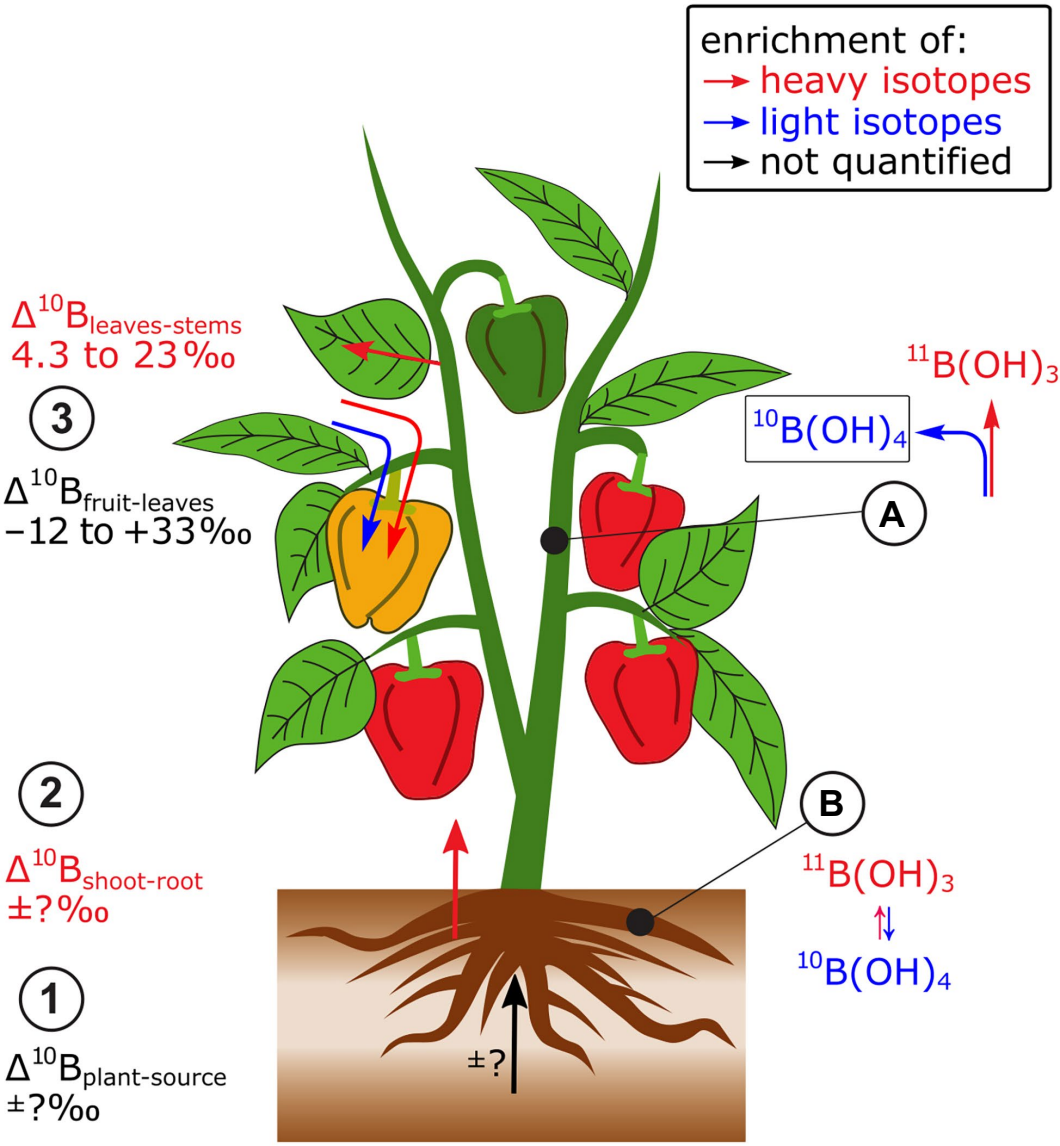
A few studies have investigated B isotope fractionation in plants. Bell pepper has been most systematically investigated. (1) Due to lacking B mass balances, Δ^11^B_plant-source_ is not known, but it can be semi-quantitatively shown that (2) shoots are enriched in heavy isotopes compared to roots. (3) Within plants, leaves are enriched in heavy B isotopes compared to stems while leaves can be heavier and lighter than reproductive organs. **(A)** At equilibrium, boric acid (B(OH)_3_) is isotopically heavier than borate (B(OH)_4_^−^) which may contribute to the isotope fractionation between root and shoot. **(B)** The integration of borate into cell walls may cause the enrichment of heavy isotopes in leaves compared to stems.

Only a few studies have systematically investigated B isotope fractionation in plants. As full mass balances were not available, only semi-quantitative information on isotope fractionation between source and plant (Δ^11^B_plant-source_), and within the plant (Δ^11^B_within.plant_) can be extracted from these data. Nevertheless, B isotope analyses in B sources, such as hydroponic nutrient solutions and plants indicated that Δ^11^B_plant-source,_ may differ between plant species or between cultivars of the same species, as demonstrated for different grapevine cultivars (Marentes et al., 1997; Coetzee et al., 2010; Figure 2). These differences are possibly linked to distinct B demand at similar B supply. In bell pepper, heavy B isotopes were preferentially transported from roots to leaves (Δ^11^B_leaves-roots_ +0.8 to +27‰, Geilert et al., 2015, 2019). Within the shoots, leaves were isotopically heavier than stems and fruits were in most cases lighter than leaves.

A main driver of B isotope fractionation within the plant may be changes of the B species boric acid (B(OH)_3_) and borate (B(OH)_4_^−^, Geilert et al., 2019; Figure 2). At equilibrium, boric acid (B(OH)_3_) is enriched in heavy isotopes compared to borate (B(OH)_4_^−^, Zeebe, 2005). Boron is transported into the root cytoplasm under B-deficient growth conditions as boric acid by the so-called Nodulin26-like Intrinsic Protein (NIP)-type membrane channels (Yoshinari and Takano, 2017). In the cytoplasm, boric acid is partially transformed to borate, which is effluxed by borate transporter proteins (BORs) membrane transporters to the xylem (Parker and Boron, 2013). Hence, the transport of B from root-to-shoot may favor the transport of light B isotopes. However, this was not the case (Geilert et al., 2019), indicating that isotope fractionation between boric acid and borate was masked by other processes. The B species are also relevant to structural binding of B into cell walls. Borate is thought to function as the structural ester “bridge” to interlink Rhamnogalacturonan-II monomers within the pectin fraction of primary cell walls (Yoshinari and Takano, 2017). Therefore, light B isotopes should be integrated into cell walls, if the integration itself does not fractionate B isotopes, while heavy B isotopes remain soluble and mobile (Geilert et al., 2015, 2019). Hence, the integration of B into cell walls could explain the enrichment of heavy isotopes in leaves compared to stems (Geilert et al., 2019).

### Magnesium

**Figure 3:**
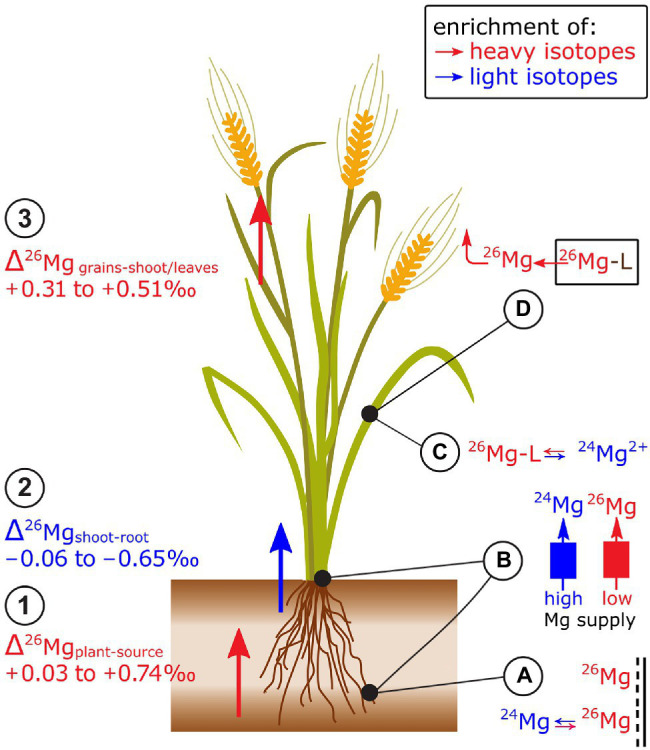
Mg isotope fractionation in plants has been studied in trees and cereals. Exemplified for cereals: (1) Plants are enriched in heavy isotopes compared to the Mg source while (2) shoots tend to be enriched in light isotopes compared to roots. (3) Grains are enriched in heavy isotopes compared to stems and leaves. **(A)** Heavy Mg isotopes preferentially bind onto negatively charged surfaces in the root apoplast. **(B)** Binding of Mg to membrane transporters that are active at low Mg supply may induce a shift towards heavy isotopes during plant uptake and root-to-shoot transport compared to Mg transport at regular (or high) Mg supply. **(C)** It is assumed that the root contains a heavy Mg pool that is bound to organic ligands (Mg-L) while ionic Mg^2+^ is enriched in light isotopes that may be preferentially transported toward the shoots. **(D)** During grain filling, organic ligands that contain Mg degrade (e.g., chlorophyll) and release heavy Mg which may lead to a preferential re-translocation of heavy isotopes from senescent tissues.

Plants preferentially take up heavy isotopes from their Mg sources, such as hydroponic nutrient solutions (Black et al., 2008; Bolou-Bi et al., 2010; Wrobel et al., 2020), phlogopite (Bolou-Bi et al., 2010), soil pore water (Tipper et al., 2010; Kimmig et al., 2018), and phytoavailable soil Mg pools (Bolou-Bi et al., 2012; Opfergelt et al., 2014; Kimmig et al., 2018; Wang et al., 2020; Figure 3). In ryegrass and clover, root extracts (CaCl_2_) of Mg revealed that heavier Mg is preferentially adsorbed to the apoplast (Bolou-Bi et al., 2010). This Mg adsorption likely contributes to the preferential uptake of heavy Mg isotopes into plants (Bolou-Bi et al., 2010; Wang et al., 2020). In wheat, Δ^26^Mg_plant-source_ was more positive under low compared to regular Mg supply (Wang et al., 2020). This difference was ascribed to distinct cross-membrane transport systems. At low supply, active Mg-specific proteins facilitate Mg uptake, while at high supply passive channels facilitate Mg uptake. The former may shift the plant Mg toward a heavier isotope composition through binding to the membrane protein (Bolou-Bi et al., 2010; Wang et al., 2020).

Shoots of grasses and clover tended to be lighter than their roots (Black et al., 2008; Bolou-Bi et al., 2010; Gao et al., 2018; Wang et al., 2020; Figure 3). Bolou-Bi et al. (2010) ascribed the negative Δ^26^Mg_shoot-root_ values to two different Mg pools in roots: ionic Mg (favoring light isotopes) and Mg bound to organic ligands (favoring heavy isotopes), such as ATP and proteins. Ionic Mg is thought to be preferentially transported from roots to shoots, leading to an enrichment of light isotopes in shoots compared to the roots. In wheat, the Δ^26^Mg_shoot-root_ was more negative in the initial growth phases (Δ^26^Mg_shoot-root_ −0.85‰) than in the final growth stages (Δ^26^Mg_shoot-root_ −0.25‰) at regular Mg supply (Wang et al., 2020). The inverse was the case for wheat grown with low Mg supply (Δ^26^Mg_shoot-root_ initial −0.25‰, final −0.55‰). Similar as for root uptake, the distinct Δ^26^Mg_shoot-root_ was ascribed to distinct cross-membrane transport modes at low and high Mg supply.

Mg isotope ratios also vary between different organs of plant shoots (Figure 3). In wheat and rice, grains were heavier than the remaining shoot (Black et al., 2008; Gao et al., 2018). However, Mg in wheat ears was lighter than the shoots at regular Mg supply, but heavier than shoots at low Mg supply (Wang et al., 2020). In spruce trees, Mg in older needles was lighter than in young needles (Δ^26^M_old-young_ of −0.30‰, Bolou-Bi et al., 2012) while Mg in old leaves of sugar maple tended to be heavier than young leaves (maximum Δ^26^Mg_old-young_ of 0.20‰, Kimmig et al., 2018). These studies indicated that Mg isotope fractionation within shoots may be related to Mg storage during vegetative growth and the subsequent remobilization from these Mg pools during reproductive growth stages. The remobilization of Mg would require a dissociation of Mg from its major organic ligands, such as chlorophyll (Kleczkowski and Igamberdiev, 2021). Theoretical and experimental studies revealed that Mg bound to chlorophyll is isotopically heavier than the bulk leaf or ionic Mg (Black et al., 2007; Moynier and Fujii, 2017; Pokharel et al., 2018; Wrobel et al., 2020). Only one study found that Mg in chlorophyll is lighter than the bulk leaf Mg (Black et al., 2008). Based on these findings, it was suggested that isotopically distinguishable pools of Mg exist in leaves, such as a light ionic Mg pool and a heavy Mg pool stored in chlorophyll (Pokharel et al., 2018). The latter may become a phloem source of Mg during leaf senescence and thereby an export of the heavier chlorophyll-bound Mg pool toward phloem sinks. However, Mg pools other than ionic and chlorophyll-bound exist in leaves and Mg isotopes bound to different organic ligands may be enriched in heavy and light isotopes compared to aqueous ionic Mg (Schott et al., 2016).

### Silicon

**Figure 4:**
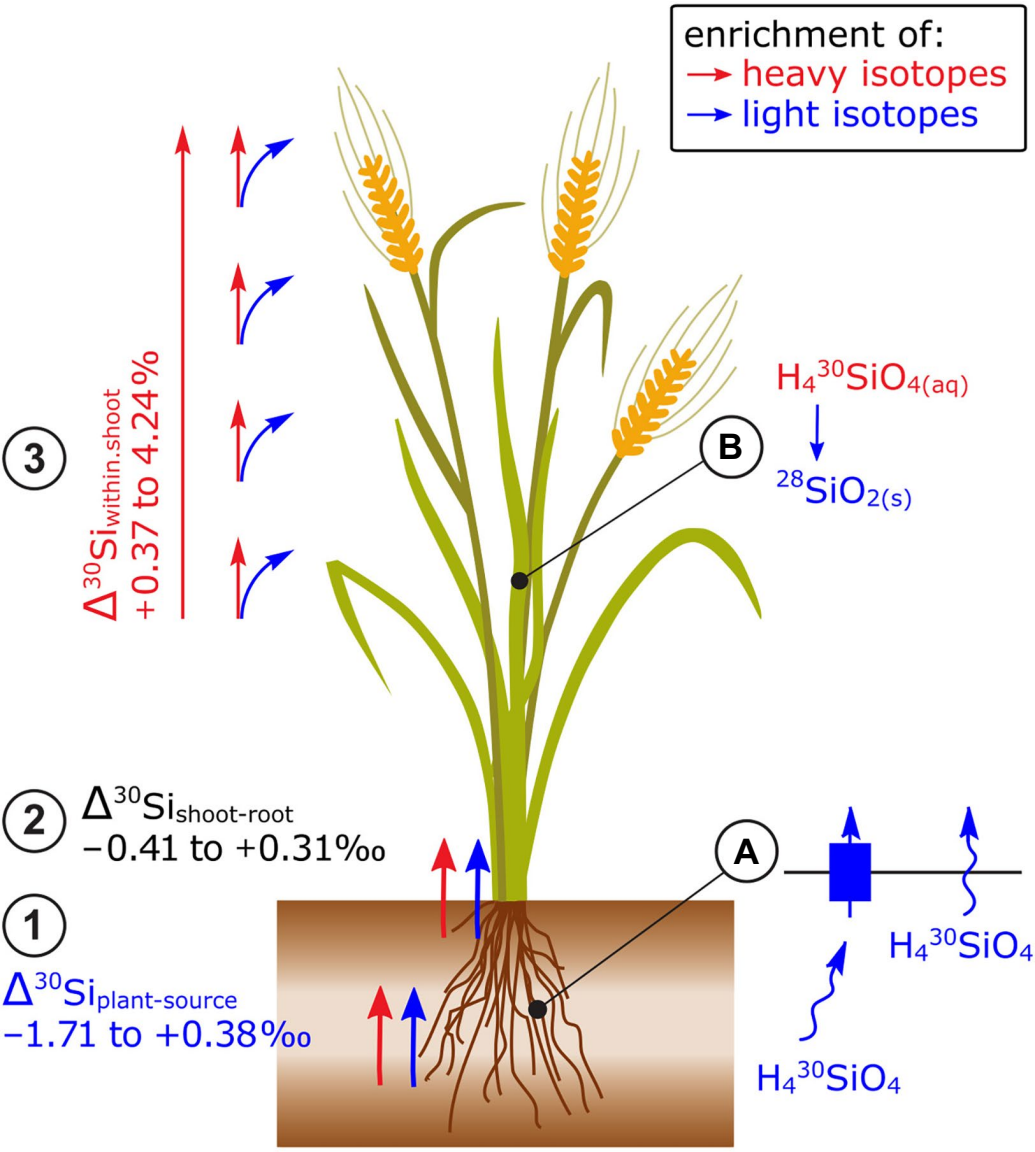
Si isotope fractionation in plants exemplified for cereals. (1) Root uptake of Si leads in most cases to an enrichment of light isotopes while (2) no consistent pattern of Si isotope fractionation has been observed between root and shoot. (3) Within the shoot, plants become significantly enriched in heavy isotopes along the transpiration stream leading to a strong enrichment of heavy isotopes in husks and grains of rice. **(A)** The preferential uptake of light isotopes has been ascribed to a non-membrane protein-mediated transfer of Si (non-Si accumulators) and a membrane protein-mediated transfer of Si (LSi1) in Si-accumulating plants. Both processes may be driven by Si diffusion that favors light Si isotopes. **(B)** Precipitation of light Si isotopes into phytoliths (SiO_2_) results in an enrichment of heavy isotopes in the Si pool (H_4_^30^SiO_4_) and thereby in an enrichment of heavy isotopes along the transpiration stream.

Most plants take up light Si isotopes from hydroponic nutrient solutions and phytoavailable soil pools (Figure 4). Frick et al. (2020) showed that light Si was preferentially taken up by Si-accumulating wheat and non-Si-accumulating tomato and mustard plants. To identify the role of membrane proteins on Si isotope fractionation, Sun et al. (2016a) reduced the biologically mediated Si uptake of rice plants by metabolic inhibitors and by cooling the hydroponic nutrient solution. These treatments reduced the Si uptake 3–4 times and induced a shift toward light isotope uptake compared to the control plants (−0.29‰). In mutant rice plants with non-functional Si membrane channel proteins (Lsi1), the Si concentration in rice shoots was 25 times reduced while Δ^30^Si in the shoots did not change (Köster et al., 2009). The enrichment of light isotopes in Si-accumulating and non-Si-accumulating plants was ascribed to diffusion during the membrane passage of Si with or without transport by membrane proteins, respectively (Frick et al., 2020). Note that Si is taken up as uncharged Si(OH)_4_ and can therefore diffuse through the cell membrane (Ma, 2015).

No consistent pattern for Δ^30^Si_shoot-root_ has been found, for example, for the same plant species (e.g., rice, −0.23 to 1.3‰, Ding et al., 2008; Sun et al., 2016a,b; Figure 4) or among non-Si-accumulating plants (−0.37 to +0.72‰, Frick et al., 2020). The processes that may govern the isotope fractionation between root and shoot are the precipitation of Si as phytoliths in roots and xylem loading (Sun et al., 2016b; Frick et al., 2020). The precipitation of Si as phytoliths favors light isotopes (Sun et al., 2016b). For xylem loading, Si needs to cross a membrane either by membrane diffusion (favors light isotopes) or by the membrane transporter Lsi2. Unlike Lsi1, Lsi2 is located at the proximal side of root exodermis and endodermis, effluxes Si out of the cell and, as a carrier protein, requires energy which is provided by the electrochemical proton gradient (Ma, 2015). A knockout of Lsi2 in rice led to a shift toward light isotopes (of ~0.5‰) in the shoots compared to the wild-type plant (Köster et al., 2009) indicating that Lsi2 may favor heavy isotopes.

Shoot organs of rice including stems, leaves, and husks become systematically enriched in heavy isotopes along the transpiration stream (Ding et al., 2005, 2008; Sun et al., 2016a; Figure 4). Similar patterns were observed for different shoot organs in banana, bamboo, and cucumber (Opfergelt et al., 2006; Ding et al., 2008; Sun et al., 2016b) and within leaves of banana and rice (Opfergelt et al., 2006; Ding et al., 2008). There, the lightest δ^30^Si were found in petioles, followed by a successive enrichment of heavy Si toward leaf tips. The systematic Si isotope fractionation within shoots was explained by a successive precipitation of light isotopes into phytoliths (Dupuis et al., 2015). The precipitation likely leads to an enrichment of heavy isotopes in the soluble Si fraction that is then further transported along the transpiration stream. In the end points of the transpiration stream, such as rice husk and grains, only a small fraction of the total Si is stored but this fraction is most enriched in heavy isotopes (Sun et al., 2016b). This successive enrichment of heavy isotopes through Si precipitation lead to a Rayleigh-like fractionation (Sun et al., 2008, 2016b). Deviations from the Rayleigh fit could be attributed to membrane protein-facilitated transport (Lsi6) within the shoot (Sun et al., 2016b).

### Calcium

**Figure 5:**
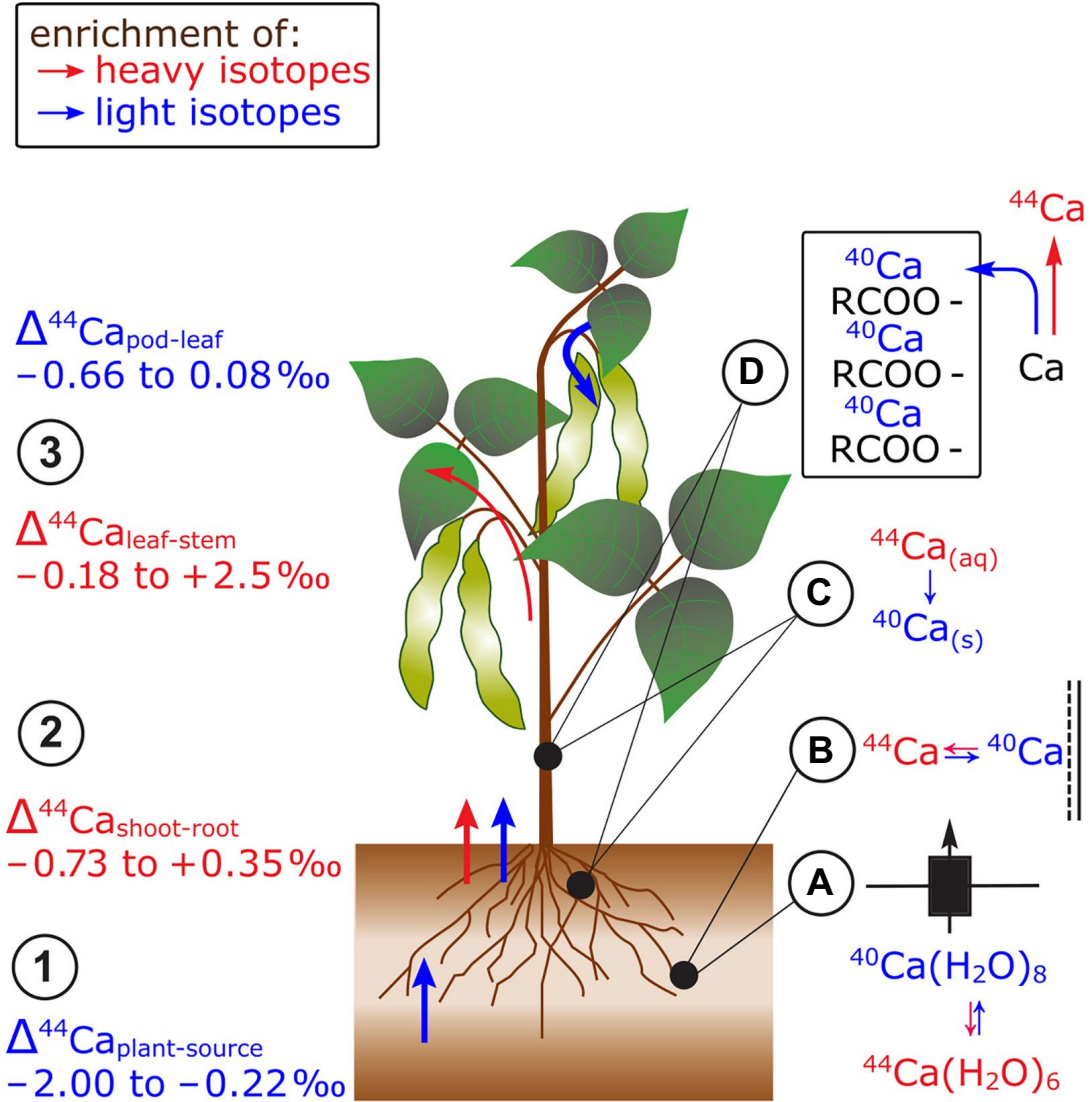
Ca isotope fractionation in plants exemplified for beans. (1) Root uptake of Ca leads to an enrichment of light isotopes. (2) In most cases, shoots are enriched in heavy isotopes compared to roots. (3) Leaves are preferentially enriched in light isotopes compared to stems while reproductive organs are mostly lighter than leaves. The preferential uptake of light isotopes has been ascribed to **(A)** isotope differences in hydrated Ca species with distinct number of water molecules and **(B)** the preferential sorption of light Ca isotopes to negatively charged surfaces in the root apoplast. Within the plant, **(C)** lighter isotopes may precipitate with oxalate (^40^Ca) and **(D)** are structurally bound in cell walls to pectins (RCOO^−^ groups). The retention of light Ca isotopes leads to a higher mobility of heavy Ca isotopes within plants.

In studies that focused on Ca cycling in catchments, Ca in trees (spruce, beech, pines) was lighter compared to its sources (Wiegand et al., 2005; Page et al., 2008; Cenki-Tok et al., 2009; Holmden and Bélanger, 2010; Bullen and Chadwick, 2016; Figure 5). These observations were confirmed in hydroponic studies on soybean, wheat, rice, and beans (Cobert et al., 2011; Schmitt et al., 2013; Christensen et al., 2018). In studies that provided full mass balances, Ca in beans and alpine plants was lighter than Ca in nutrient solutions or bulk soils, respectively (Δ^44/40^Ca_plant-source_ −0.23 to −1.03‰, Hindshaw et al., 2013; Schmitt et al., 2013). The enrichment of light Ca isotopes during root uptake was assigned to preferential binding of light Ca to negatively charged surfaces, such as pectines in the root apoplast (Cobert et al., 2011; Schmitt et al., 2017, 2018). The fractionation between nutrient solution and beans could be modeled using a constant equilibrium fractionation factor between free ionic Ca and Ca sorbed to pectin groups (Δ^44/40^Ca_pectine-ionic_ −0.12‰, Schmitt et al., 2013). In addition, Ca channel-mediated membrane transport may fractionate Ca isotopes as the transport rate of Ca ions may be size specific. The effective size of Ca ions depends on the number of water molecules in the first hydration shell and less hydrated Ca ions tend to be isotopically light (Moynier and Fujii, 2017).

There is no systematic fractionation of Ca isotopes between roots and shoots of different species (Hindshaw et al., 2013; Schmitt et al., 2013; Christensen et al., 2018; Figure 5). By employing a sequential extraction procedure for roots, Schmitt et al. (2018) showed that 90% of Ca in the roots of beech trees stabilizes cell walls and membranes as Ca-pectate. This structural Ca was lighter than the water soluble Ca fraction, suggesting that light Ca isotopes were retained in the roots while heavy isotopes were transported to the shoot. Hindshaw et al. (2013) explained the retention of light Ca isotopes in roots with Ca precipitation as oxalate in the root cortex of alpine herb. The assumption that Ca oxalate is enriched in light Ca isotopes was confirmed by extraction of Ca oxalate from leaves (Cobert et al., 2011; Schmitt et al., 2018). However, no profound explanation exists yet for the favorable transport of light isotopes from roots to shoots in, for example, soybean (Christensen et al., 2018).

Leaves tended to be heavier than stems in cereals, beans, and alpine plants (Page et al., 2008; Cobert et al., 2011; Hindshaw et al., 2013; Schmitt et al., 2013, 2017; Christensen et al., 2018; Figure 5). This fractionation might be explained by successive removal of light Ca from the xylem sap into cell walls of the xylem tissues (Cobert et al., 2011; Schmitt et al., 2018). Therefore, heavy Ca is further transported along the transpiration stream toward the leaves. Furthermore, pods of beans were lighter than leaves but not distinguishable from stems at regular Ca supply (Cobert et al., 2011; Schmitt et al., 2013). However, the isotope fractionation between leaves and pods was negligible at low Ca supply which strongly suggests that different processes controlled the Ca transfer to the pods at distinct Ca supply. Despite the fact that Ca is usually considered as phloem immobile, Ca mass balances revealed that Ca can be re-translocated from roots and leaves of several plant species at low Ca supply (Maillard et al., 2015). Hence, the degree of Ca re-translocation in plants could determine the Ca isotope composition in phloem sinks and sources.

### Iron

**Figure 6:**
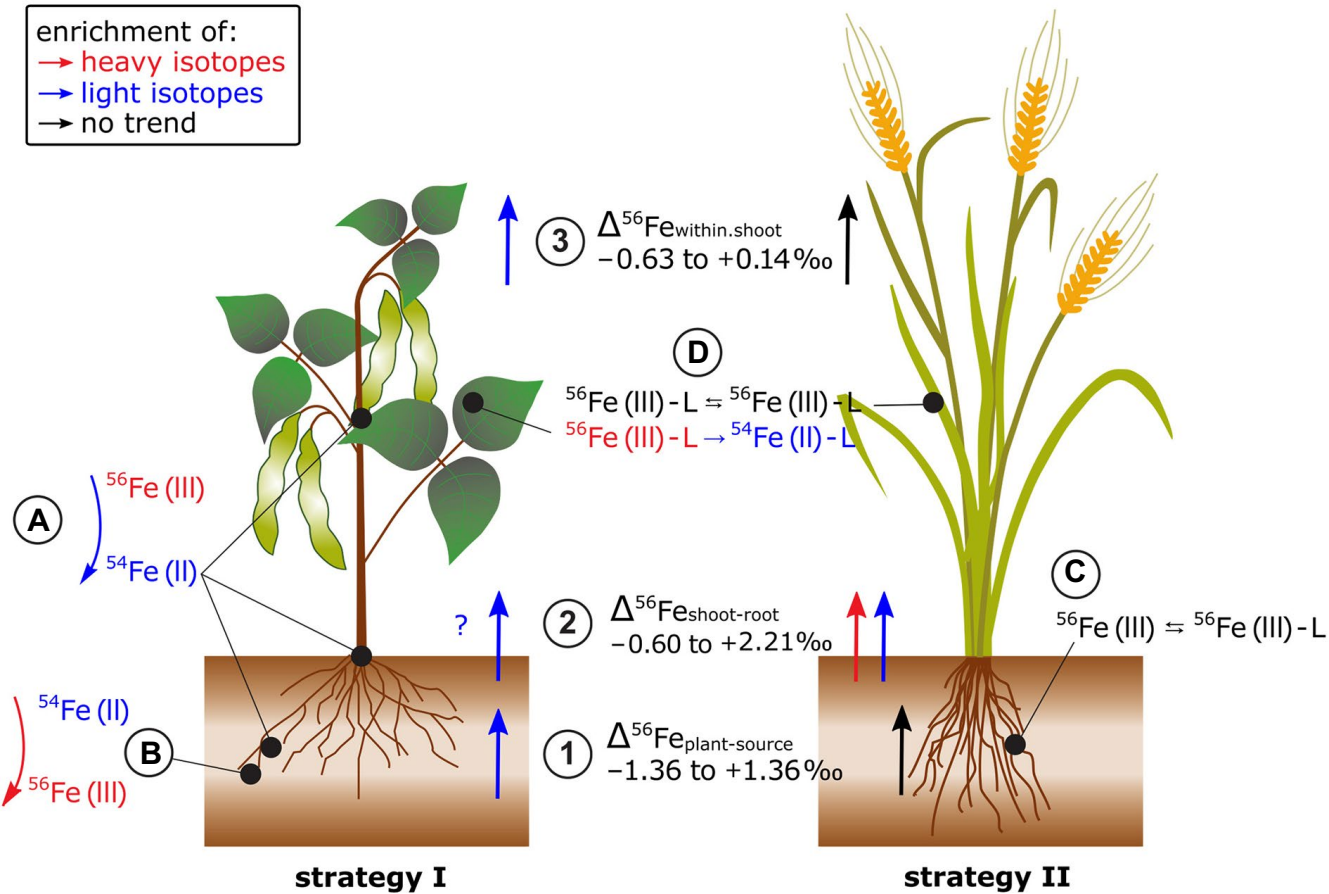
Fe isotope fractionation in plants for Fe acquisition strategy I and II plants. (1) Fe isotope fractionation between the Fe source and the plant strongly depends on the Fe source (i.e., Fe-EDTA in hydroponics, bulk soil, or soil extract). In most cases, Fe acquisition by plants leads to a stronger enrichment of light isotopes in strategy I plants (e.g., tomato and beans) compared to strategy II plants. (2) The Fe isotope fractionation between shoot and root highly depends on the root Fe pools analyzed since significant fractionation can occur between precipitated Fe in the root apoplast compared to Fe that is taken up into the symplast and not deposited in the root apoplast. (3) Reproductive organs tend to be enriched in light isotopes in strategy I plants while no such fractionation step occurs in strategy II plants. **(A)** The Fe reduction from Fe(III) to Fe(II) prior to membrane transport may explain the successive enrichment of light isotopes from soil *via* roots and leaves to reproductive organs in strategy I plants. **(B)** Fe reoxidation from soluble Fe(II) to insoluble Fe(III) in the root apoplast can lead to an enrichment of light Fe isotope in the soluble Fe fraction. **(C)** In strategy II plants, the mobilization of Fe(III) from the mineral lattice of the soil by phytosiderophores (L for ligand, e.g., deoxymugineic acid) does not require a Fe reduction step and leads, if at all, to small Fe isotope fractionation. **(D)** Within the shoot, ligand (L) exchange of Fe (e.g., nicotianamine, citrate, and ferritin) that can include Fe redox changes may further fractionate Fe isotopes.

Plants preferentially take up heavy Fe from phytoavailable soil pools (Δ^56^Fe_plant-phytoavailable_ −0.11 to +1.36‰, Garnier et al., 2017; Wu et al., 2019; Liu et al., 2019; Chen et al., 2021; Figure 6). Plant Fe acquisition from soils includes the dissolution of Fe(III) minerals and root membrane transport, but the Fe acquisition strategy differs among plant species (Connorton et al., 2017). Strategy I plants (e.g., tomato) are equipped with root surface reductases to reduce insoluble Fe(III) to Fe(II) prior to cross-membrane transport. Strategy II plants (e.g., oat) release phytosiderophores that mobilize Fe(III) from the mineral lattice. The Fe(III)-phytosiderophore complex is then transported across the plasma membrane. The isotope fractionation Δ^56^Fe_plant-bulk.soil_ was 1.8‰ lighter in strategy I (e.g., bean) compared to strategy II (e.g., oat) plants (Guelke and von Blanckenburg, 2007). In strategy I plants, kinetically controlled reduction of Fe(III) to Fe(II) prior to membrane transport could yield large isotope fractionation (Johnson et al., 2020). In strategy II plants, Fe binding to chelating molecules released by the plant may induce comparably small equilibrium Fe isotope fractionation (Dideriksen et al., 2008). These results were confirmed in hydroponics where Fe was supplied as Fe(III)-EDTA (Guelke-Stelling and von Blanckenburg, 2012). The negative isotope fractionation during uptake was more pronounced in strategy I (bean) compared to strategy II (oat) plants. Strategy I plants may have reduced Fe(III)-EDTA to Fe(II) while no reduction was required during the ligand exchange from Fe(III)-EDTA to Fe(III)-phytosiderophores in strategy II plants (Guelke-Stelling and von Blanckenburg, 2012; Liu et al., 2019). Hence, the absence and presence of a Fe reduction step might be the major factor that causes distinct Fe isotope fractionation in strategy I and II plants during Fe acquisition.

Several studies indicated that the distinct Δ^56^Fe_plant-source_ values of strategy I and II plants are not universally applicable (Kiczka et al., 2010; Moynier et al., 2013; Liu et al., 2019; Wu et al., 2021; Figure 6). In field studies, the Δ^56^Fe_plant-source_ values of strategy I and II plants differed less. This is probably due to less controlled environmental conditions in the field and/or the use of a variety of non-model plants for which Fe acquisition strategies are less known. In addition, there might be other processes than root surface reduction and chelation by phytosiderophores that cause Fe fractionation during plant acquisition. In paddy soil-rice systems, root plaque is formed in the oxidized rhizosphere where Fe precipitates as Fe(III) oxides in the root apoplast (Garnier et al., 2017; Chen et al., 2021). This precipitation induced a strong isotope fractionation between pore water and root plaque (Δ^56^Fe_root.plaque-pore.water_ +1.41 to +2.24‰). Moreover, Fe isotope composition of roots differed with and without Fe plaque. Similar findings were also reported for plants grown in aerated soils for different Fe pools in the roots of alpine plants (Kiczka et al., 2010). These results highlight that Fe (re-)precipitation processes in the rhizosphere and apoplast need to be considered to approach the “true” Δ^56^Fe_plant-source_ value.

Iron isotope fractionation between root and shoot (Δ^56^Fe_shoot-root_) ranged from −0.60 to +2.02‰ (Kiczka et al., 2010; Guelke-Stelling and von Blanckenburg, 2012; Wu et al., 2019, 2021; Chen et al., 2021; Figure 6). There was no clear preference for light or heavy Fe isotope transport from root to shoot while different methodologies to separate Fe root pools limit the comparability of the Δ^56^Fe_shoot-root_ data (see previous paragraph).

Fe isotopes fractionate significantly between different shoot organs (Guelke and Von Blanckenburg, 2007; Guelke-Stelling and von Blanckenburg, 2012; Arnold et al., 2015; Chen et al., 2021; Figure 6). Δ^56^Fe_within.shoot_ differed between strategy I (e.g., beans) and II plants, similar as for Δ^56^Fe_plant-source_ (e.g., oat, Guelke-Stelling and von Blanckenburg, 2012). Seeds of strategy I plants were lighter than the remaining shoot while no such fractionation occurred in strategy II plants or rice (Guelke and Von Blanckenburg, 2007; Guelke-Stelling and von Blanckenburg, 2012; Arnold et al., 2015; Chen et al., 2021). The preferential transport of light Fe isotopes into seeds in strategy I was ascribed to Fe reduction (Guelke-Stelling and von Blanckenburg, 2012). Within the leaf cells, heavier isotopes are thought to be preferentially stored as Fe(III) in the vacuoles or in proteins, such as ferritin. The remobilization of Fe requires a reduction of Fe(III) to Fe(II) for transport as, for example, Fe(II)-nicotianamine. Likewise, the negligible Fe isotope fractionation within shoots of strategy II plants indicates that Fe(III) is either quantitatively reduced to Fe(II) or such redox changes are absent.

### Nickel

**Figure 7:**
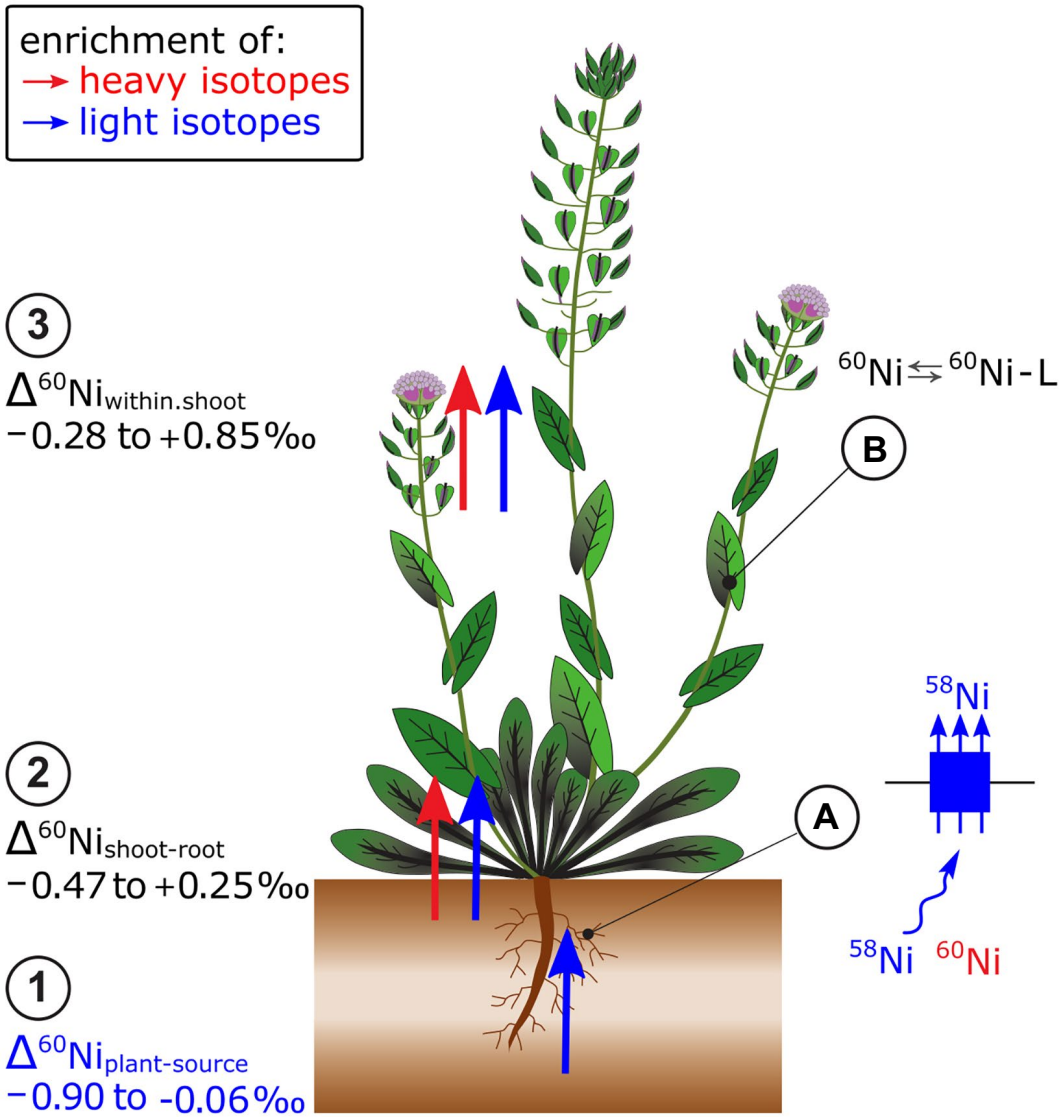
Nickel isotope ratios have been mostly measured in Ni hyperaccumulating plants. (1) Root uptake leads to an enrichment of light Ni isotopes in plants. (2) From roots to shoots as well as (3) within shoots, though no clear pattern of isotope fractionation has been identified so far. **(A)** It is hypothesized that the enrichment of light isotopes during plant uptake of Ni is induced by low-affinity transport. High Ni uptake rates in hyperaccumulator plants may induce a depletion of Ni in the proximity of the membrane transporters and induce a Ni diffusion which leads to additional enrichment of light isotopes during uptake. **(B)** Within the shoot, Ni speciation to organic ligands (L), such as citrate, may be excluded as major factor controlling Ni isotope fractionation.

Plants that grew on soils with high Ni concentrations were isotopically lighter than phytoavailable soil pools (Δ^60^Ni_plant-extract_ −0.51 to −0.06‰, Estrade et al., 2015; Zelano et al., 2020; Figure 7). In hydroponics, light isotopes were also preferentially taken up from the nutrient solution (Δ^60^Ni_plant-solution_, −0.90 to −0.07‰, Deng et al., 2014). Nickel hyperaccumulator plants took up lighter isotopes compared to Ni non-hyperaccumulator plants (Deng et al., 2014). In hyperaccumulators, the Ni uptake may be kinetically controlled due to the high metal transport rate of low-affinity transporters that leads to depletion of Ni in the rhizosphere. The Ni flow from bulk solution toward the roots may be diffusion driven which favors light isotopes (Rodushkin et al., 2004). Besides, Ni speciation in the hydroponic nutrient solution may have induced isotope fractionation between the free ionic Ni^2+^ and the complexed Ni pool. Particularly at low Ni supply, only a small fraction of Ni was present as free ionic Ni, while most of the Ni was complexed to EDTA. The EDTA may have enriched the free ionic Ni fraction in light isotopes and thereby contributed to the uptake of light Ni isotopes in plants. Heavy Ni isotope complexation to organic ligands was corroborated by theoretical and experimental studies on Ni-citrate complexes (Fujii et al., 2014; Zelano et al., 2020).

The Δ^60^Ni_shoot-root_ and Δ^60^Ni_within.shoot_values were positive, negative or zero, depending on the plant and growth conditions (Deng et al., 2014; Estrade et al., 2015; Ratié et al., 2019; Zelano et al., 2020; Figure 7). In hydroponically grown plants, a much higher fraction of Ni was transported from roots to shoots in hyperaccumulators, compared to the non-hyperaccumulator. In these experiments, the isotope fractionation followed distinct patterns: hyperaccumulator plants had negative Δ^60^Ni_shoot-root_ values, whereas those of non-hyperaccumulators were positive (Deng et al., 2014). Both Δ^60^Ni_shoot-root_ and Δ^60^Ni_leaves-stem_ varied with plant age and phenological stage of plants (Estrade et al., 2015; Zelano et al., 2020). The biochemical processes that generate the variation in Δ^60^Ni_shoot-root_ and Δ^60^Ni_leaves-stem_ are not well understood except that experimentally derived equilibrium fractionation factors as well as combined speciation and isotopes studies suggest that Ni complexation to organic ligands in plants is not a main driver of Ni isotope fractionation in plants (Zelano et al., 2018, 2020).

### Copper

**Figure 8:**
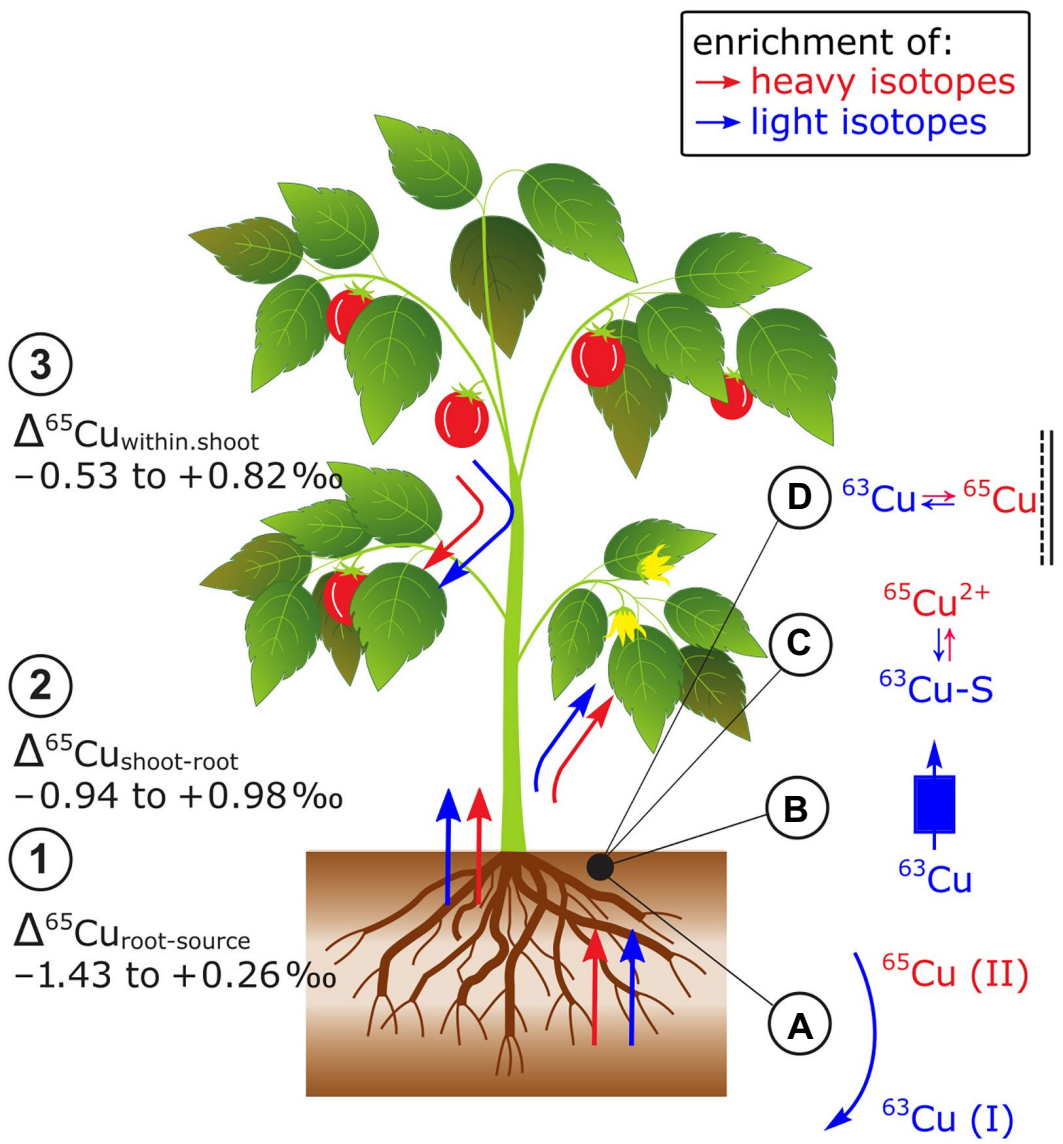
Cu isotope fractionation in plants exemplified for tomato plants. (1) Root uptake can lead to an enrichment of heavy or light Cu isotopes. The isotope fractionation during root uptake is more negative in Fe strategy I plants compared to strategy II plants. At low Cu supply light isotopes were preferentially taken up compared to heavy isotopes at high supply likely due to a shift from active to passive uptake. (2) Shoots can be enriched in light and heavy isotopes compared to roots. The enrichment of light isotopes increases with organ height and Cu availability. (3) Cu isotopes fractionate in shoots of Fe strategy I plants is stronger than in strategy II plants. **(A)** Cu reduction induced by root reductases favors light Cu isotopes, **(B)** Cu membrane transport can favor light Cu isotopes, and **(C)** Cu binding to S to detoxify Cu in the root at high Cu supply may contribute to the retention of light isotopes in roots. **(D)** Cu binding to O ligands in the root apoplast should retain heavy Cu in the roots.

Cu isotope fractionation between the Cu source and plants (Δ^65^Cu_root-source_) and the Cu source and the root can be positive, negative, or zero (Jouvin et al., 2012; Ryan et al., 2013; Li et al., 2016; Blotevogel et al., 2022; Figure 8). Results on Cu isotope fractionation during plant uptake are difficult to compare as in most cases Δ^65^Cu_roots-source_ values were reported instead of Δ^65^Cu_plant-source_. Furthermore, plants were exposed to distinct Cu sources in hydroponics (e.g., ionic Cu^2+^ vs. chelated Cu) and Cu bound to the root apoplast was desorbed in one study prior to root analyzes (Ryan et al., 2013). Nonetheless, preferential uptake of light isotopes seems to be controlled by the reduction of Cu(II) to Cu(I) prior to root membrane transport into tomato (Jouvin et al., 2012; Ryan et al., 2013). The Cu reduction may explain why strategy I plants (according to their Fe acquisition strategy, see section Iron) take up lighter isotopes than strategy II plants. For the latter, the Cu reduction seems to be not a requirement for Cu uptake (Ryan et al., 2013). However, studies on yeast mutants showed that specific Cu membrane transporters favor light isotopes without a preceding Cu reduction indicating that the preferential uptake of light Cu isotopes is not solely controlled by Cu reduction (Cadiou et al., 2017). Moreover, distinct Fe supply neither affected Δ^65^Cu_plant-source_ nor Δ^65^Cu_roots-source_ (Jouvin et al., 2012; Ryan et al., 2013) while a higher Cu supply shifted Δ^65^Cu_roots-source_ from light to heavy in grapevine plants (Blotevogel et al., 2022). This shift was assigned to a switch from active toward passive uptake pathways due to increased Cu availability. Together, the identified processes that control Δ^65^Cu_plant-source_ are Cu reductase activity at the root surface, membrane transport, and adsorption to root surfaces that may all vary with Cu supply.

Copper isotope fractionation between root and shoot (Δ^65^Cu_shoot-root_) can be positive, negative or zero (Weinstein et al., 2011; Jouvin et al., 2012; Ryan et al., 2013; Li et al., 2016; Blotevogel et al., 2022; Figure 8). Similarly, as for the Δ^65^Cu_plants-source_ values, the comparison of Δ^65^Cu_shoot-root_ values may be limited particularly due to different Cu root desorption strategies (Jouvin et al., 2012; Ryan et al., 2013). Enrichment of light isotopes in shoots compared to roots was ascribed to Cu diffusion and membrane transport that were categorized as kinetic fractionation processes (Weinstein et al., 2011; Jouvin et al., 2012). Light isotope enrichment in leaves was accentuated in grapevine at high Cu supply (Blotevogel et al., 2022). Exposed to high Cu concentrations and after root Cu desorption, Δ^65^Cu_shoot-root_ was neutral in oat (strategy II) while tomato (strategy I) shoots were enriched in heavy isotopes compared to their roots (Ryan et al., 2013). The contrasting Δ^65^Cu_shoot-root_ in Fe strategy I and II plants were ascribed to the reoxidation of Cu(I)-S to Cu(II) prior to xylem loading (Ryan et al., 2013). Similar to Fe, this reoxidation step may only occur in strategy I plants which results in a strong enrichment of heavy isotopes in the shoots compared to the roots. However, in strategy II plants, the reoxidation step might be absent or all Cu(I) is reoxidized to Cu(II).

Within plant shoots, an enrichment of light isotopes in leaves appears to increase with plant height in hairy-leaved sedge (Weinstein et al., 2011) and/or with Cu concentration in grapevine leaves (Blotevogel et al., 2019, 2022; Figure 8). The enrichment of light Cu isotopes was ascribed to diffusion and membrane transport (Weinstein et al., 2011) as well as Cu immobilization by Cu complexation to S (Cu(I)-S) that favors light isotopes (Cadiou et al., 2017; Blotevogel et al., 2019). However, detailed analyses of the Cu tolerant plant *E. splendens* revealed that redistribution of Cu from senescent to younger leaves and reproductive organs can cancel the correlations between leaf height and Cu concentration with Cu isotope ratios (Li et al., 2016). Ryan et al. (2013) found a more pronounced Cu isotope fractionation between stems and leaves in Fe strategy I than in II plants. Similarly to Fe, the stronger isotope fractionation in strategy I plants is likely driven by Cu redox cycles while these cycles may not control the Cu isotope fractionation in shoots of strategy II plants.

### Zinc

A previously published review summarized that adsorption, type of membrane transport (low vs. high-affinity transport), speciation, compartmentalization, and diffusion control the Zn isotope fractionation in plants (Caldelas and Weiss, 2017). This conclusion was based on studies that investigated a diversity of plant species and applied methods, such as (i) Zn uptake studies with unicellular organisms (Gélabert et al., 2006; John et al., 2007), (ii) root extraction techniques (Tang et al., 2016), (iii) elaborated hydroponic (e.g., Smolders et al., 2013) and pot studies (Houben et al., 2014; Couder et al., 2015), (iv) combined Zn speciation and isotope analyses (Aucour et al., 2015), and (v) a set of theoretically and experimentally determined isotope fractionation factors for Zn binding to organic ligands (Jouvin et al., 2012; Fujii et al., 2014; Marković et al., 2017).

**Figure 9:**
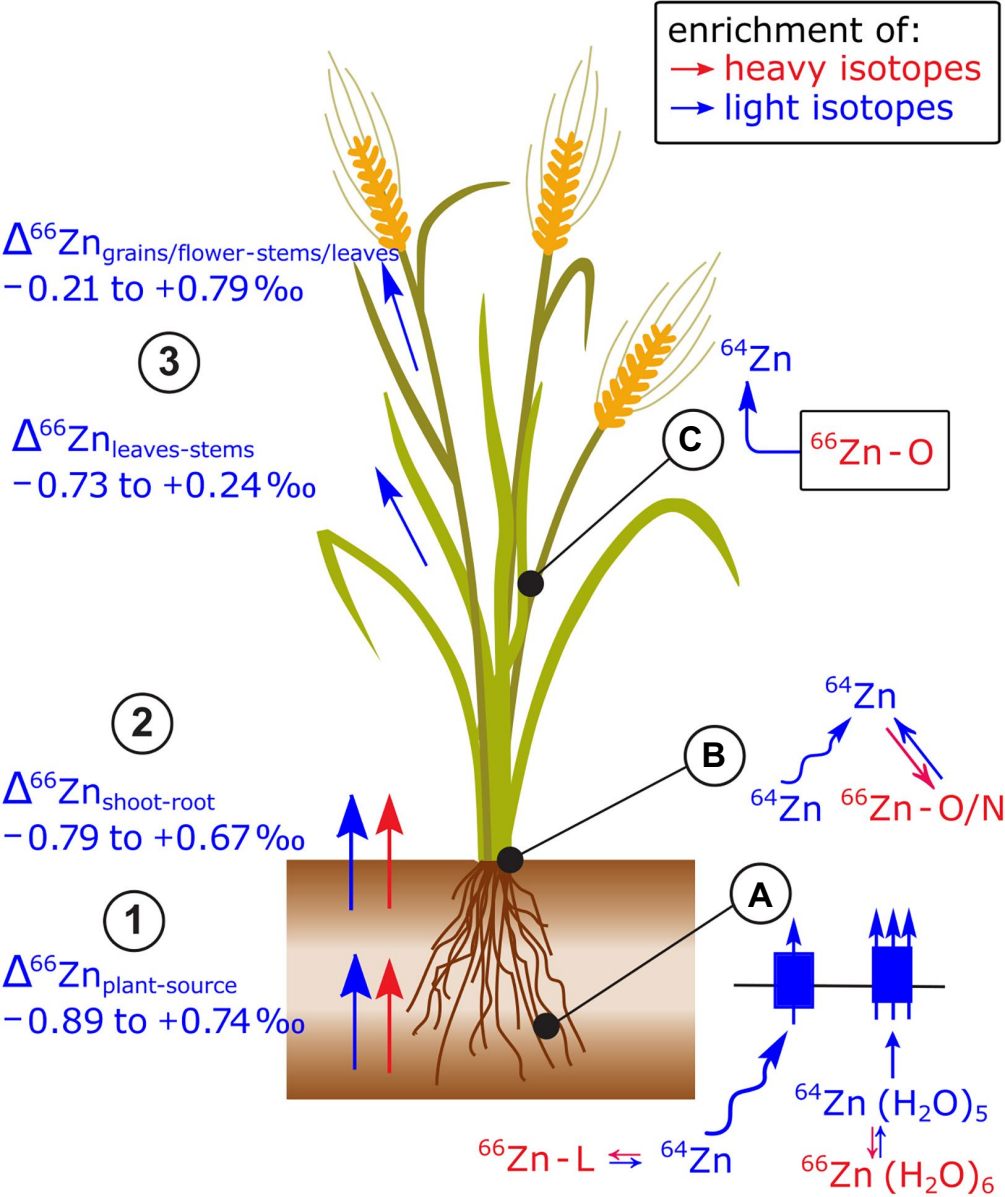
Zn isotope fractionation has been reported in several plant species (see review of Caldelas and Weiss, 2017 and references therein). (1,2) In most cases, light Zn isotopes are preferentially taken up by plants and transported from root to shoot. (3) Within the shoots, leaves are enriched in light isotopes compared to stems and reproductive organs are enriched in light isotopes compared to the remaining shoot or senescent tissues. **(A)** The uptake of light Zn isotopes has been ascribed to diffusion, Zn speciation in solution, and stripping of the hydration shell prior to membrane transport. A set of studies provides robust evidence, that at low Zn supply, Zn complexation to organic ligands (L), such as phytosiderophores followed by the uptake of the Zn-phytosiderophores leads to a shift toward heavy isotopes in cereals. During membrane transport, the enrichment of light isotopes is stronger at regular than at low Zn supply. **(B)** Within the root, binding of Zn to O/N donors of organic ligands in the cytosol and vacuole as well as diffusion of the Zn in the apoplast and symplast toward the xylem may control the preferential transport light isotopes from roots to shoots. **(C)** The enrichment of light Zn isotopes in reproductive plant organs is induced by the strong retention of heavy isotopes in mature leaves or senescing tissues likely induced binding of Zn to O donors of organic ligands in the apoplast, cytosol, or vacuole.

After the review of Caldelas and Weiss (2017); a field study on paddy soil-rice systems showed more positive Δ^66^Zn_rice-source_ values at low Zn than at regular Zn supply (Weiss et al., 2021; Figure 9). This shift toward heavy Zn isotopes at low Zn supply can be explained by chelators (e.g., the phytosiderophores 2’deoxymugineic acids) that are secreted by roots to strip Zn from the soil matrix and subsequent uptake of the entire Zn-phytosiderophore complex by a membrane transporter (Smolders et al., 2013; Arnold et al., 2015; Weiss et al., 2021). Since 2’deoxymugineic acids bind heavy Zn isotopes at equilibrium (Marković et al., 2017), the uptake of Zn-phytosiderophore complexes may have induced the shift toward heavy isotopes. Furthermore, a unicellular organism study showed a dependence of Δ^66^Zn_cell-source_ on Zn uptake rates (Köbberich and Vance, 2017). At low uptake rates, heavier Zn isotopes were taken up than at high uptake rates. This study is discussed in detail in section Heavy but More Mobile and the Question of Kinetic vs. Equilibrium Fractionation that focuses on unicell studies.

Changes to Zn isotope composition in the shoot of wheat (Δ^66^Zn_within.shoot_) were measured during the grain filling period (Wiggenhauser et al., 2018). In this period, leaves and stems showed a net loss of Zn and a depletion of light isotopes, while grains became enriched in light isotopes compared to stems and leaves. These results strongly suggest that light Zn isotopes were transported within the phloem toward the grains while heavy isotopes were retained in senescing shoot organs. The retention of heavy Zn isotopes in senescent shoot organs was ascribed to Zn binding to O containing ligands in the apoplast, such as pectins (Aucour et al., 2017).

### Cadmium

**Figure 10:**
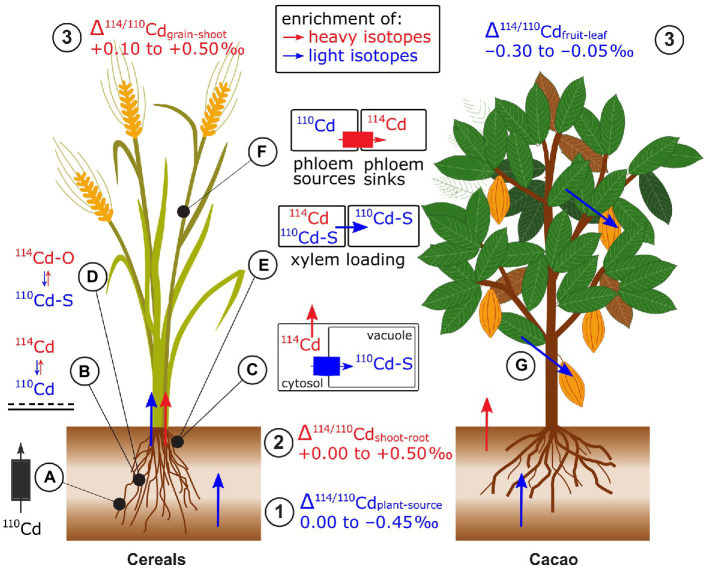
Cadmium isotope fractionation in plants exemplified for cereals and cacao. (1) Root uptake leads to an enrichment of light isotopes in plants. (2) Shoots are mostly enriched in heavy isotopes compared to roots. (3) In cereals, grains are enriched in heavy isotopes compared to the remaining shoot while in cacao, the beans tend to be enriched in light isotopes compared to other shoot parts. **(A)** Root membrane transport of NRAMP5 induces an enrichment or depletion in light Cd isotopes during uptake. **(B)** Light isotopes are preferentially sorbed to the negatively charged surfaces in the root apoplast. **(C)** Light Cd isotopes are sequestrated in root vacuoles *via* tonoplast proteins. **(D)** Chelation of Cd by thiols (Cd-S) contributes to the sequestration of light Cd in roots. **(E)** The non-membrane bound protein CAL1 that preferentially binds light Cd with thiols in the xylem parenchyma cells and transports light Cd into the xylem. **(F)** Xylem to phloem transfer in the nodes favors heavy Cd isotopes through transport by OsHMA2 and LCT. **(G)** In cacao, given that beans are enriched in light isotopes compared to leaves, the processes transporting and loading Cd into beans may differ from cereals.

Plants were isotopically lighter than their phytoavailable Cd source, in hydroponic and soil studies (Wei et al., 2018; Imseng et al., 2019; Moore et al., 2020; Wiggenhauser et al., 2021a; Zhang et al., 2021; Zhong et al., 2021, 2022; Figure 10). An exception was that a hyperaccumulator plant grown on Cd contaminated soil had a heavier isotope composition than the phytoavailable pool (+0.02 to +0.18‰, Zhou et al., 2020). Two rice studies found that the enrichment in light isotopes was slightly enhanced in non-flooded compared to flooded soils (Wiggenhauser et al., 2021a; Zhong et al., 2022) while one found the opposite (Zhang et al., 2021). Possible factors causing this difference may be distinct initial soil properties (e.g., pH and soil Cd concentration) or changes in the phytoavailability of elements, such as Mn and Fe upon flooding. Several studies investigated the role of NRAMP5 on Cd isotope fractionation during uptake. In rice (*Oryza sativa*), both a small upregulation and knockout of *OsNRAMP5* were associated with uptake of lighter Cd isotopes compared to control treatments (Zhang et al., 2021; Zhong et al., 2022). This apparent discrepancy is discussed in section Genetic Approaches for Isotope Fractionation Factors. Yeasts expressing cacao (*Theobroma cacao, Tc*) *TcNRAMP5* preferentially took up lighter Cd isotopes compared to the control yeast (i.e., with empty vector, Moore et al., 2020). In addition to membrane transport, light Cd uptake may be partly due to adsorption of preferentially light Cd onto the root apoplast (Δ^114/110^Cd_root absorbed-adsorbed_ of −0.17‰, Zhang et al., 2021).

In most cases, shoots were isotopically heavier than roots in wheat, barley, rice, cacao, and a Cd accumulator plant (Wiggenhauser et al., 2016, 2021a,b; Wei et al., 2018; Imseng et al., 2019; Moore et al., 2020; Wang et al., 2021; Zhong et al., 2021; Figure 10). The retention of light Cd isotopes in roots of cereals and numerous different cacao genotypes was ascribed to vacuolar sequestration. This explanation is supported by Rayleigh isotope fractionation models (Imseng et al., 2019; Moore et al., 2020). Furthermore, while fitting the same isotope model, substantial differences in translocation of Cd were observed for distinct cacao clones, which was ascribed to differences in the expression of genes that encode dominant Cd transporters, rather than distinct biochemical processes (Moore et al., 2020). The role of vacuolar sequestration on Cd isotope fractionation was further supported by results of wild-type mutant experiments on rice (Zhang et al., 2021). These results suggested that the tonoplast transporter heavy metal ATPase 3 (HMA3) preferentially transports light Cd isotopes into the vacuole. In the vacuole, Cd is then sequestered by strong binding to thiols that may contribute further to the retention of light Cd (Wei et al., 2018). This hypothesis is supported by synchrotron X-ray absorption spectroscopy (XAS) results that showed the majority of Cd in rice roots can be bound to S-containing ligands, such as glutathione or phytochelatins, which are expected to preferentially bind light Cd isotopes (Yan et al., 2016; Wiggenhauser et al., 2021a; Zhao et al., 2021). Alternatively, Zhong et al. (2022) showed that Δ^114/110^Cd_shoot-root_ in rice can be further impacted by the protein CAL1. This protein complexes Cd in xylem parenchyma cells and Cd-CAL1 complexes are then transported into the xylem. Furthermore, the higher expression of *CAL1* coincided with a strong shift toward light isotopes during root to shoot translocation.

Cd isotope composition in reproductive and shoot organs can strongly differ (Δ^114/110^Cd_reproductive-shoot.organ_ −0.30 to +0.50‰, Figure 10). While Rayleigh fractionation modeling for different plants suggests dominantly unidirectional xylem flow from roots to shoots (Wiggenhauser et al., 2016; Wei et al., 2018; Moore et al., 2020), it has been shown that cereals likely use phloem redistribution on grain filling (Wiggenhauser et al., 2021b; Zhong et al., 2021). In rice, an important hub for the transfer of Cd from the xylem to the phloem are the nodes (Yamaji and Ma, 2014). First data on Cd isotopes in nodes indicated that the xylem to phloem transfer of Cd contributes to the enrichment of heavy isotopes in grains (Wiggenhauser et al., 2021b; Zhong et al., 2021). Additionally, combined isotope and gene expression analyses strongly suggested that membrane proteins (OsHMA2, OsLCT1) that transfer Cd from the xylem to the phloem in the node contribute to the enrichment of heavy Cd in grains (Zhong et al., 2021). The role of Cd speciation in the nodes (mainly Cd-S) on Cd isotope fractionation between nodes, leaves, and grains is not understood yet (Wiggenhauser et al., 2021b). In contrast to cereals, Cd loading into cacao beans may use a different mechanism (Barraza et al., 2019) since Cd in cacao beans was found to be isotopically lighter than in the leaves. The identical isotope compositions between cacao leaves and leaf litter support this hypothesis, because isotope fractionation would be expected if there was phloem redistribution on senescence.

### Emerging Elements

#### Potassium

Soybeans and grasses preferentially took up light isotopes from hydroponics (Δ^41/39^K_plant-solution_ of −0.60‰) and also from phytoavailable soil pools (Christensen et al., 2018; Li et al., 2021). Δ^41/39^K_shoots-roots_ were reported to be positive and negative, however, full mass balances were not provided. Within shoots, dead leaves were isotopically lighter than living leaves (Li et al., 2021). During plant uptake and translocation, membrane transport may strongly control K isotope fractionation (Christensen et al., 2018). The selectivity of an ion channel is at least partly controlled by the ionic radius and could explain the preferential uptake of ^39^K over ^40^K due to the smaller ionic radius of ^39^K (Lockless et al., 2007). Additionally, K transport through an ion channel requires dehydration which would favor ^39^K for membrane transport (MacKinnon, 2003; Hofmann et al., 2012; Christensen et al., 2018). Within the plant, diffusion may only play a minor role for K isotope fractionation in moving saps, such as in the xylem and phloem (Christensen et al., 2018). Binding of heavy isotopes to pectate may be an additional factor that controls the K isotope fractionation in plants, as deduced from combined K isotope and XAS analysis (Li et al., 2021).

#### Molybdenum

The first Mo isotope analyses in plants revealed that in three out of four plant species (lingonberry, common juniper, and rosebay willowherb), heavy isotopes were preferentially transported from roots to stems (Δ^98^Mo_stem-root_ −0.00 to +0.70‰) and from stems to leaves (Δ^98^Mo_leaves-root_ −0.00 to +0.40‰), while in blueberry, Mo isotopes were not fractionated (Malinovsky and Kashulin, 2018). No processes have been yet ascribed to the systematic Mo isotope fractionation patterns.

#### Thallium

White mustard grown in hydroponics preferentially took up light isotopes (Δ^205^TI_plant-source_ −0.20 to −0.09‰) while heavy isotopes were transported from roots to shoots, particularly at high Tl supply (Δ^205^Tl_shoot-root_ −0.07 to +0.72‰, Vaněk et al., 2019). Within shoots, Tl isotope fractionation reached Δ^205^Tl_shoot-root_ of 0.43‰ (Kersten et al., 2014; Rader et al., 2019; Vaněk et al., 2019). Although there is no known biological function for Tl, it has been posited that the isotope fractionation between roots and shoots is due to physiologically controlled translocation which may be related to membrane transport and Tl speciation. Since Tl^+^ and K^+^ have similar ionic radii (1.76 Å and 1.60 Å), it has also been suggested that Tl uses K channels to translocate (Vaněk et al., 2019). In addition, Tl in plants appears to be mostly present as Tl(I)aq and Tl-acetate (O-ligand) in roots, stems, and leaves.

## Discussion

Linkages of biological and physico-chemical processes that are known to fractionate isotopes in plants, and highlights knowledge gaps that could be effectively filled, are summarized in Supplementary Table S3. In the next sections, we interlink the information collected for the individual elements in the results section to advance the use of non-traditional isotopes for process tracing in plants.

### Similar Elements, Similar Isotope Fractionation?

Chemically similar elements can undergo similar or opposite isotope fractionation in plants. For example, for the redox sensitive transition metals Fe and Cu, reduction can be required prior to membrane transport (Cadiou et al., 2017; Johnson et al., 2020, Figures 6; 8). The reduction enriches the reduced Fe and Cu fractions in light isotopes and may largely control the shift toward light isotopes during acquisition in strategy I plants (Guelke-Stelling and von Blanckenburg, 2012; Ryan et al., 2013).

In contrast, for the non-redox sensitive transition metals Zn and Cd, the within-plant isotope fractionation differs as light Zn but heavy Cd isotopes are more mobile in plants (Arnold et al., 2015; Wiggenhauser et al., 2018; Figures 9, 10). Currently, two hypotheses are discussed that explain the opposing isotope fractionation between Zn and Cd. The first is that the distinct isotope fractionation is caused by the higher affinity of Cd to S donors of organic molecules (e.g., cysteine) compared to Zn, which in turn has a higher affinity to O and N donors (e.g., histidine) compared to Cd (Maret and Moulis, 2013). Thiol chelators (e.g., phytochelatin) can strongly bind Cd and contribute to the sequestration of light Cd isotopes in vacuoles (Nocito et al., 2011). Based on *ab initio* calculations, preferentially light Zn and Cd isotopes bind to S chelators at equilibrium (e.g., Fujii et al., 2014; Zhao et al., 2021). Given that Cd has a higher affinity to thiols than Zn, competition for thiol binding could control the opposing isotope fractionation of these trace metals in cereals. The second hypothesis is based on Cd sorption experiments on humic acid and *ab initio* calculations (Ratié et al., 2021; Zhao et al., 2021). Binding of ionic Cd to carboxyl groups (Cd-O) leads to a small shift toward light isotopes (Ratié et al., 2021). The isotope shift was ascribed to the dehydration of Cd from Cd(H_2_O)_6_ to Cd(H_2_O)_5_ prior to binding of Cd to carboxyl groups. The dehydration of Cd favors light isotopes while the opposite is the case for Zn (Fujii et al., 2014; Zhao et al., 2021). Hence, the opposing isotope fractionation of Zn and Cd could be induced by dehydration processes that may take place prior to membrane transport or during ligand exchange and/or by the high affinity of Cd to thiols.

Similarly, the alkaline earth metals Ca and Mg fractionate in an opposite manner during plant uptake (Figures 3, 5). This is partly due to preferential sorption of light Ca and heavy Mg isotopes onto negatively charged root surfaces (Bolou-Bi et al., 2010; Schmitt et al., 2017). Therefore, plants preferentially take up lighter Ca but heavier Mg isotopes compared to their Ca and Mg source. In a study on goethite, which like pectins provides negatively charged O donors, Mg had a tendency to form inner-sphere complexes, resulting in the loss of its water coordination shell. Calcium, in contrast, formed outer-sphere complexes, keeping its water coordination shell (Rahnemaie et al., 2006). Hence, the opposing isotope fractionation of Ca and Mg can likely be explained by a difference in the stability of their hydration sphere (Essington, 2015), as also hypothesized for Zn and Cd. The within-plant isotope fractionation of Ca and Mg does not clearly differ (Figures 3, 5). Hence, processes, such as structural binding to cell walls or oxalate precipitation for Ca, and cross-membrane transport and partitioning in functional molecules for Mg, may mask isotope effects induced by dehydration and sorption.

These examples illustrate that specific chemical properties can induce marked differences in isotope fractionation in plants, even if elements belong to the same group in the periodic table. Hence, the use of different elements to explain isotope fractionation patterns by analogy is limited.

### Heavy but More Mobile and the Question of Kinetic vs. Equilibrium Fractionation

For elements, such as Mg, Si, and Cd, heavy isotopes are more mobile than light isotopes in plants (Figures 3, 4, 10). These findings contradict the established idea that biological processes in plants favor the transport of light isotopes due to enzymatic reactions that are kinetically controlled (Dawson et al., 2002). Preferential uptake of heavy Mg isotopes seems to be controlled by the sorption to root surfaces (Figure 4). In this case, equilibrium isotope fractionation seems to outcompete membrane transport, which may be kinetically controlled. Light Si isotopes precipitate in roots as phytoliths and light Cd isotopes are likely sequestered in vacuoles, leading to higher mobility of heavy isotopes within the plant (Figures 4, 10; Ding et al., 2009; Wiggenhauser et al., 2021a). Precipitation of Si in shoots is likely kinetically controlled because it takes place at the end of the transpiration stream, where water evaporation leads to constant supersaturation and new Si influx. In contrast, vacuolar sequestration may be caused by a mix of kinetic and equilibrium fractionation (membrane transport and Cd chelation). These examples illustrate that kinetically controlled processes do not necessarily lead to a higher mobility of light isotopes in plants.

The importance to distinguish between kinetic and equilibrium fractionation was highlighted for Zn uptake of marine diatoms. These unicell organisms preferentially took up heavier Zn isotopes at low supply but light isotopes at high Zn uptake rates (John et al., 2008; Köbberich and Vance, 2017). At high Zn uptake rates, the negative Δ^66^Zn_organism-source_ was ascribed to kinetic effects during membrane transport and to Zn speciation in the nutrient solution. At low Zn uptake rates, a pseudo-equilibrium may have been established between the ionic Zn in solution and the binding site of the membrane transporter, leading to a shift toward heavy isotopes (Jouvin et al., 2009; Fujii et al., 2014). These interpretations suggest that isotope fractionation of one biological process may not in all cases be kinetically or equilibrium controlled. Instead, there might be environmental conditions in which only kinetic, only equilibrium or both, determine the isotope fractionation in biological organisms. Deciphering whether isotope fractionation is kinetic or equilibrium is of high priority to advance process tracing by non-traditional isotope systems in plants.

The general rule that biological processes in plants favor the transport of light isotopes due to enzymatic reactions is based on studies on traditional isotopes, such as C, N, and S. For instance, the enzyme rubisco that catalyzes CO_2_ fixation in plants induces a kinetically controlled enrichment of light C isotopes during C assimilation (O’Leary, 1993). Similarly, enzymatic processes involving N assimilation, such as nitrate reductase or glutamine synthetase, favor light N isotopes (Tcherkez, 2011). However, metals and metalloids act as cofactors for enzymes rather than being the target element of complex metabolism pathways as with C, N, and S. Yet, enzyme controlled processes can be crucial for the homeostasis of redox sensitive metals, such as Fe and Cu or the Mg-dechelatase mediated decomposition of chlorophyll (Connorton et al., 2017; Chen et al., 2018; Zandi et al., 2020). Hence, the impact of enzymatically controlled processes on the fractionation for non-traditional isotopes in plants might differ from traditional isotope systems.

### Unicellular Organisms: A Suitable Model System for Isotope Studies?

Studies of unicellular organisms (unicell) have proven to be an effective tool to gain a detailed understanding of the biological uptake of nutrients and pollutants. Yeast mutants provided evidence that the reduction of Cu(II) to Cu(I), controlled by the reductase FRE1 and FRE2, leads to light Cu isotope enrichment during uptake (Cadiou et al., 2017). However, the mutants with dysfunctional reductases were still enriched in light isotopes, indicating that the high-affinity Cu transporters CTR1 and CTR3 preferentially transport light Cu isotopes. This illustrates that the negative Δ^65^Cu_cell-source_ is driven by both reduction and membrane transport. The yeast study confirmed previously stated hypotheses that Cu reduction leads to a shift toward light isotopes during root uptake into strategy I plants, such as tomato (Jouvin et al., 2012; Ryan et al., 2013).

Isotope fractionation during uptake and compartmentalization of Cd was investigated using transgenic yeasts and *E. coli* (Horner et al., 2013; Moore et al., 2020). In yeast transformed with cacao (Tc) genes, expression of *TcNRAMP5* was associated with uptake of light Cd isotopes. These findings supported the hypothesis that the preferential binding of light Cd to the membrane transporter contributes to the overall enrichment of light Cd isotopes in cacao plants (Figure 10). Although the growth conditions were similar for both organisms, the magnitude of the fractionation was larger for the yeast suggesting that other transporters are contributing significantly to Δ_organism-source_ in cacao. Horner et al. (2013) measured Cd isotopes in cell walls and other compartments of *E. coli*. The cell walls were enriched in light isotopes which was ascribed to Cd binding to cell wall thiols. Similarly to *E. Coli*, Cd isotopes in cereal roots were also enriched in light isotopes possibly due to vacuolar sequestration and detoxification through binding of Cd to chelating thiols in root vacuoles of cereals (Wiggenhauser et al., 2021a).

Cyanobacteria were employed to determine Mg isotope fractionation during uptake and compartmentalization (Pokharel et al., 2018). The positiveΔ_organism-source_ value for cyanobacteria was related to Mg binding to functional molecules, such as chlorophyll or ATP in the cells. This binding generated intracellular Mg pools that were enriched in heavy (bound Mg) and light Mg isotopes (ionic Mg). Mass balances showed that the light ionic Mg pool was likely transported out of the cell, leading to a net enrichment of heavy Mg isotopes in cyanobacterium and thereby to a positive Δ_organism-source_. This intracellular Mg isotope fractionation may explain negative Mg^26^Δ_shoot-root_ found in plants (Figure 3) where Mg bound to functional molecules (heavy) is retained in roots while ionic Mg (light) is transported through the xylem into the shoot. Likewise, heavy Mg isotopes stored in functional molecules in leaves could be re-translocated from senescing plant organs *via* phloem toward reproductive plant organs. However, such a relation was not found in wheat sampled at different growth stages (Wang et al., 2020). Hence, Mg binding to functional molecules alone does not explain the changing isotope compositions in leaves, which may be affected by Mg import and export in leaves and/or by long-distance transport pathways *via* xylem and phloem.

Together, unicell studies can provide a powerful method to test specific hypotheses on potential fractionation mechanisms in plants and to identify specific isotope fractionation factors. However, results of unicell studies need to be carefully extrapolated to plants as plants have developed several strategies to acquire elements (e.g., regulation of several membrane transporters, root exudation) and are complex organisms that exchange elements between different cells, tissues, and organs.

### Genetic Approaches for Isotope Fractionation Factors

A variety of genetic approaches have been used to determine isotope fractionation factors associated with individual membrane proteins. For example, three different techniques have been used to investigate how NRAMP5 impacts the fractionation of Cd during uptake in cacao and rice (unicell transgenics, gene knockout, and gene expression monitoring). A yeast experiment using a gene from cacao (TcNRAMP5) supported the hypothesis that TcNRAMP5 preferentially transports light Cd isotopes (see section Heavy but More Mobile and the Question of Kinetic vs. Equilibrium Fractionation) and thereby contributes to the enrichment of light isotopes in cacao plants. Conversely, the knockout experiment in rice found that rice without *OsNRAMP5* preferentially took up light Cd compared to its wild type (Zhang et al., 2021). Since plants have a suite of different membrane proteins that can also transport Cd, the result favoring the interpretation that OsNRAMP5 takes up preferentially heavy Cd may reflect the upregulation of genes encoding other membrane proteins that induce an even larger isotopic shift than OsNRAMP5. The two gene expression experiments found that *OsNRAMP5* in rice was moderately upregulated in non-flooded soil (Zhong et al., 2021, 2022). In Zhong et al. (2022), there was no significant difference in Δ^114/110^Cd_rice-source_ between flooded and non-flooded conditions, while Zhong et al. (2021) found an enrichment in light isotopes in rice that grew on the non-flooded conditions where *OsNRAMP5* was upregulated. Although the experimental conditions and the soil-rice systems of these two experiments were identical, the causes of these distinct observations were not discussed. Nevertheless, the studies of Zhong et al. (2021, 2022) are in agreement with the transgenic experiment results, which strongly suggest that NRAMP5 can contribute to preferential uptake of light isotopes in plants. The comparisons show that complementary techniques (e.g., knockout and transgenics), at similar environmental conditions, are necessary to cross-validate results and interpretations. The distinct outcomes of the NRAMP5 studies highlight that carefully obtained isotope fractionation factors with controlled model systems may still be masked by environmental factors in complex soil–plant systems.

## Future Perspectives

Isotope fractionation in plants is usually very systematic and can differ, for example, among plant ages, species, and nutrient supply. Although biotic and abiotic factors induce systematic isotope fractionation in plants, its interpretation is often challenging and relies on basic knowledge in plant sciences. Here we provide recommendations to better exploit the information provided by varying isotope compositions in plants to further advance non-traditional isotope process tracing in plants. Once these recommendations are implemented, isotope process tracing could be applied to detect and quantify changes in uptake and translocation pathways in response to changing nutrient supply as suggested for Mg (Wang et al., 2020), Cu (Blotevogel et al., 2022), and Zn (Weiss et al., 2021).

### Basic Requirements for Plant Isotope Experiments

An indispensable requirement for the interpretation of isotope fractionation patterns in plants is the establishment of fractionation factors for specific physico-chemical processes. This requires experiments to quantify isotope fractionation for individual processes, such as precipitation (e.g., Guinoiseau et al., 2018) or binding to organic ligands (e.g., Ratié et al., 2021). In addition, there is a set of minimum requirements that must be taken into account to ensure a meaningful data set and discussion.

Planning phase:

- design experiments to test specific hypotheses and determine (co-)variables. These can range from environmental conditions, such as (i) pH (Cobert et al., 2011), (ii) varying nutrient sources (Rader et al., 2019), or (iii) nutrient limitations (Smolders et al., 2013), to biological changes, such as wild-type–mutant experiments (Zhang et al., 2021),
- include biological replicates to account for experimental variability (Wang et al., 2020),
- conduct plant growth experiments at physiologically relevant conditions to ensure nutrient levels are not additional variables impacting the isotope signatures of the targeted element,
- thoroughly document growth conditions (e.g., nutrient levels, light, temperature, plant protection regime), specific tissue type, and phenological growth stages at harvest (Meier, 2001).

Sample preparation:

- consider root treatments at harvest, such as root desorption by weak salt solutions (Tang et al., 2016). This separates apoplastic and symplastic element pools and enables the determination of isotope fractionation for apoplastic sorption and cross-membrane transport,
- take into account that the phytoavailability of elements and isotope composition in bulk and rhizosphere soil can be very different (e.g., Imseng et al., 2019).

Data analysis and evaluation:

- calculate the average concentration and isotopic mass balance of target elements. This is needed to elucidate fractionation upon root uptake from growth media (the Δ_root-solution_ does not inform on the fractionation during root uptake),
- once isotope compositions in different plant organs are measured, geochemical modeling approaches (e.g., Moore et al., 2020) can be used and incorporated with existing plant science knowledge to interpret what biological and physico-chemical processes are responsible for the systematic isotope fractionation patterns,
- analyze concentrations of other elements that may compete with the element of interest for, for example, membrane transporter and organic ligands.

### Innovative Research Ideas and Methods

Provided that the requirements that are listed in section Basic Requirements for Plant Isotope Experiments are met, the following ideas can be included to expand the scope of plant stable isotope analyses. The ideas were selected from the individual element reviews and studies beyond those on stable isotope fractionation in plants.

- Isotope analyses in different plant organs and tissues (e.g., cortex, epidermis, rhizodermis) that are sampled at different growth stages (e.g., flowering, plant maturity) can provide useful information on internal transport processes and pathways of elements within plants (e.g., Wang et al., 2020).
- Measuring the isotope composition of different elements in the soil–plant system (e.g., Wiggenhauser et al., 2018).
- Further compartmentalization, for example, analysis of xylem sap, would provide complementary information to Δ_shoot-root_ and Δ_shoot-grain/bean_ values to understand the processes that control the transfer of elements from the roots to shoot organs including reproductive organs (Álvarez-Fernández et al., 2014).
- To aid such detailed and comprehensive experiments, improvements to sample throughput should be made. The efficiency of sample purification could be improved by (i) automated ion chromatography or sample purification systems (Husson et al., 2015; Morgan et al., 2018; Kidder et al., 2020; Zhou et al., 2021) or (ii) using new generation MC-ICP-MS instruments that are equipped with high-resolution modes, quadrupole pre-mass filters or collision/reaction cells to reduce interferences (e.g., Christensen et al., 2018; Moynier et al., 2021).
- Wild-type mutant comparisons (e.g., Zhang et al., 2021) as well as monitoring the gene expression of plants in response to different environmental conditions (Zhong et al., 2021) can be used to investigate the role of specific proteins in element storage and transport. Determining the isotope fractionation factor for specific proteins using these techniques is challenging as, for example, compensatory mechanisms, such as the abundance alteration of other proteins, may contribute to the signal (Chang et al., 2020).
- Further constraint of isotope fractionation factors associated with individual transporters can be achieved by characterization of their transport features following heterologous expression in simple systems; such as unicellular organisms like yeasts (e.g., *Saccharomyces cerevisiae*, Cadiou et al., 2017; Moore et al., 2020), insect cells or frog eggs (*Xenopus oocytes*, Larsen et al., 2017), model plant species, or the reconstitution of purified transporters in defined lipid bilayers (Xie, 2008).
- Speciation analyses can provide complementary information to isotope analyses (e.g., Li et al., 2021). Synchrotron XAS provides information on the chemical environment of the target element in plant organs (e.g., bound to O, N, or S donors or specific molecules, such as citrate).
- Combining liquid chromatography with MC-ICP-MS. This has been used to measure Mg and Cu isotope ratios in chlorophyll and proteins (Larner et al., 2019; Wrobel et al., 2020). For structural elements, such as B and Ca, extraction techniques can provide crucial insights into isotope fractionation within plants (Schmitt et al., 2018).
- Analysis of organelles, such as vacuoles and cell walls, could improve the understanding of isotope fractionation on a subcellular level in plants (Horner et al., 2013; Song et al., 2014). Such approaches require small volumes of analytes that could be facilitated by bespoke sample introduction systems (Murphy et al., 2020).
- Coupling laser ablation with MC-ICP-MS could provide *in-situ* isotope ratio analyses at tissue level (Lobo et al., 2018). This method has recently been tested to measure Si isotope ratios of phytoliths in leaves (Frick et al., 2019) and can potentially be applied for other elements.
- Finally, it would be interesting to combine isotope analyses for traditional and non-traditional elements. Nitrogen isotopes may be highly complementary to non-traditional isotopes (e.g., Mg due to its role in chlorophyll, Laursen et al., 2013). Another example is C isotope data, which can provide information on water use efficiency (Newton, 2016). Linking, for example, C and K isotope fractionation may also be highly relevant due to the role of K in controlling stomatal conductance.

## Conclusion

This review illustrates that non-traditional isotopes usually fractionate significantly and systematically in plants. The current knowledge suggests that fractionation is driven by: diffusion for Si uptake, root apoplast adsorption for Ca and Mg, membrane transport and chemical speciation for Zn and Cd, reduction of Fe and Cu, precipitation of elements into insoluble forms, such as phytoliths (Si) or oxalate (Ca), and structural binding to cell walls as shown for Ca and probably also B. These processes can induce similar (Cu, Fe) or distinct (Ca vs. Mg, Zn vs. Cd) isotope fractionation patterns for chemically similar elements. In addition, isotope compositions of the covered elements vary between plant species/varieties and in distinct environmental conditions. Hence, isotope process tracing in plants can potentially provide unique information on dominant biological and physico-chemical processes that control uptake and transport of elements in distinct soil–plant systems. For instance, it can be used to understand changes in uptake and translocation mechanisms that occur in response to changing nutrient supply, as shown for, for example, Mg, Zn, and Cu. Therefore, isotope process tracing could be a valuable tool to identify how plants cope with distinct environmental stresses and soil management strategies. Further exploitation of the scope of isotope process tracing requires the joint expertise of plant scientists and geochemists to develop hypothesis-driven experimental designs for plant growth trials, conduct robust and efficient isotope analyses, employ biological model systems, integrate contrasting environmental conditions, and to ultimately link isotope fractionation in plants to physiological processes. This expertise can only be covered by research collaborations that include (at least) plant and isotope geochemical scientists. Such interdisciplinary research may have great potential to overcome current thinking boundaries and thereby advance isotope and plant physiological knowledge.

## Author Contributions

The interdisciplinary team of plant and geo scientists including MW, RM, GB, KHL, and SB contributed to conceptualization, methodology, investigation, data curation, writing of original draft, and visualization. PW contributed to writing and editing. MW was responsible for the project administration. All authors contributed to the article and approved the submitted version.

## Conflict of Interest

The authors declare that the research was conducted in the absence of any commercial or financial relationships that could be construed as a potential conflict of interest.

## Publisher’s Note

All claims expressed in this article are solely those of the authors and do not necessarily represent those of their affiliated organizations, or those of the publisher, the editors and the reviewers. Any product that may be evaluated in this article, or claim that may be made by its manufacturer, is not guaranteed or endorsed by the publisher.
