# Supplementary material for: Stable Isotope Fractionation of Metals and Metalloids in Plants: A Review
Source: Front Plant Sci. 2022 Apr 19;13:840941. doi: 10.3389/fpls.2022.840941 (PMC9063737; doi:10.3389/fpls.2022.840941)
Supplement: Supplementary file 1 [file Data_Sheet_1.pdf]

Supplementary Information

to

Stable isotope fractionation of metals and metalloids in plants: a review

Matthias Wiggenhauser[1†*], Rebekah E. T. Moore[2†*], Peng Wang[3], Gerd Patrick Bienert[4], Kristian Holst Laursen[5], Simon Blotevogel[6]

[1]Group of Plant Nutrition, Institute of Agricultural Sciences, Department of Environmental System Science, ETH Zurich, Zurich, Switzerland

[2]MAGIC Group, Department of Earth Science and Engineering, Imperial College London, London, UK

[3]Nanjing Agricultural University, College of Resources and Environmental Sciences, Nanjing, China

[4]Crop Physiology, Molecular Life Sciences, Technical University of Munich, Freising, Germany

[5]Plant Nutrients and Food Quality Research Group, Plant and Soil Science Section and Copenhagen Plant Science Centre, Department of Plant and Environmental Sciences, Faculty of Science, University of Copenhagen, Denmark

[6] Laboratoire Matériaux et Durabilité des Constructions (LMDC), UPS/INSA, Université de Toulouse III, Toulouse, France

[†]shared first authorship

Corresponding author: Matthias Wiggenhauser (matthias.wiggenhauser@usys.ethz.ch)

Table S1: An overview of non-traditional stable isotope methods

Element (symbol)	Stable isotopes (natural abundances, %)	Ratio[a]	Analyte amount[b] ng	Separation procedures	Primary standard	Instrumentation	Isobaric interferences[f]	Mass bias correction	Analytical precision ‰
Boron<br>(B)	10 (19.9) 11 (80.1)	11/10	200-400	Geilert et al. 2015<br>Rosner et al. 2011	NIST 951<br>NIST 951a	ICP-Q-MS<br>MC-ICP-MS<br>TIMS		SSB[g]	>0.8
Magnesium<br>(Mg)	24 (78.99) 25 (10.00) 26 (11.01)	25/24<br>26/24	1000-3000	Bolou-Bi et al. 2009<br>Opfergelt et al. 2012<br>Wang et al. 2020	NIST 980<br>DSM3	MC-ICP-MS		SSB	>0.1
Silicon<br>(Si)	28 (92.230) 29 (4.683) 30 (3.087)	30/28	600-3000[c]	Schuessler & Blankenburg 2014<br>Georg et al. 2006<br>Oelze et al. 2016	NIST 8546	MC-ICP-MS<br>LA-MC-ICP-MS	$^{15}N_2$	SSB<br>Mg doping	0.10-0.11 (bulk),0.24 (*in situ*)
Potassium<br>(K)	39 (93.2581) 40 (0.012) 41 (6.730)	41/39	3000-10000	Richter et al. 2011<br>Li et al. 2016<br>Morgan et al. 2018<br>Chen et al. 2019	NIST 999b<br>NIST 3141a	MC-ICP-MS<br>(isoprobe)		SSB	0.05 to 0.20
Calcium<br>(Ca)	40 (96.941) 42 (0.647) 43 (0.135) 44 (2.086) 46 (0.004)	44/40	2000-5000	Bullen & Chadwick 2016<br>Schmitt et al. 2009<br>Christensen et al., 2018	NIST 915a	TIMS<br>MC-ICP-MS	$\mathbf{^{40}Ar}$ $^{20}Ne^{2+}$ $^{21}Ne^{2+}$ $^{22}Ne^{2+}$	Double spike SSB	0.09-0.12
Iron<br>(Fe)	54 (5.845) 56 (91.754), 57 (2.119) 58 (0.282)	57/54<br>56/54	1500-5000<br>100-500[d]	Schoenberg & von Blanckenburg 2005, Teutsch et al. 2005<br>Strelow 1980	IRMM-014	MC-ICP-MS	^{54}Cr ^{58}Ni	SSB<br>Ni/Cu-doping	0.05-0.12
Nickel<br>(Ni)	58 (68.077) 60 (26.223) 61 (1.140) 62 (3.635) 64 (0.9256)	60/58	100-200	Quitté and Oberli 2006<br>Cameron et al. 2009<br>Ratie et al. 2019	NIST 986	MC-ICP-MS	^{58}Fe	Double spike	0.05-0.10
Copper<br>(Cu)	63 (69.17) 65 (30.83)	65/63	100-300	Blotevogel et al. 2018<br>Borrok et al. 2007<br>Kidder et al. 2020	NIST 976[e]	MC-ICP-MS		SSB Ni/Zn/Ga doping	0.02-0.15
Zinc<br>(Zn)	64 (48.63) 66 (27.9) 67 (4.10) 68 (18.75) 70 (20.84)	66/64	50-200	Caldelas et al. 2020<br>Marković et al. 2017<br>Moore et al. 2017	JMC-Lyon[e]	MC-ICP-MS	^{64}Ni Ba^{2+} ^{70}Ge $^{35}Cl_2$	Double spike<br>Cu/Ni-doping	0.05-0.10
Molybdenum<br>(Mo)	92 (14.84) 94 (9.25) 95 (15.92) 96 (16.68) 97 (9.55) 98 (24.13) 100 (9.63)	98/95	20-100	Kerl et al. 2017<br>Malinovsky & Kashulin 2018	NIST 3134	MC-ICP-MS	^{92}Zr ^{94}Zr ^{96}Zr ^{98}Ru ^{100}Ru	Double spike Pd-doping	0.04-0.20
Cadmium<br>(Cd)	106 (1.25) 108 (0.89) 110 (12.49) 111 (12.80) 112 (24.13) 113 (12.22) 114 (28.73) 116 (7.49)	114/110	20-100	Murphy et al. 2016<br>Wei et al. 2015	NIST 3108	MC-ICP-MS TIMS	^{106}Pd ^{108}Pd $^{110}Pd$$^{112}Sn$ ^{113}In,^{113}In ^{114}Sn ^{116}Sn	Double spike Ag-doping SSB	0.02-0.10
Thallium<br>(Tl)	203 (29.524) 205 (70.476)	205/203	10-100	Rader et al. 2018<br>Baker et al. 2009	NIST 997	MC-ICP-MS		SSB Pb-doping	0.04-0.10

[a]Isotope ratio most commonly used to report isotope composition, usually using the delta (δ) notation relative to the primary isotope standard. Other ratios are used to assess whether isotope fractionation is mass dependent or independent.

[b]The range of analyte amount used for isotopic measurement with good analytical precision. The minimum value will provide enough for one measurement, while the maximum value is preferred, to enable analytical repeats. Note that these are indicative values and can increase or decrease depending on mass bias correction method, sensitivity (V/ppm analyte), the uptake rate of the nebuliser (MC-ICP-MS) and wet or dry plasma conditions.

[c] This is the amount used for the isotope measurement, but more (up to 60 ug) is required for the separation procedure (NaOH fusion)

[d] For Fe, measurements with lower analyte amounts can be undertaken using new generation MC-ICP-MS instrumentation

[e] No longer available so others used and then conversion applied to data. For Cu: AE633 or AE647 (Moeller et al. 2012), For Zn: AA-ETH or IRMM-3702 (Archer et al. 2017)

[f] There are also multiple possible molecular interferences including hydrides, nitrides, oxides and argides. For example, potentially problematic molecular interferences for ^{56}Fe and ^{63}Cu are $^{40}Ar^{16}O$ and $^{23}Na^{40}Ar$, respectively.
These can be found online. E.g. https://www.perkinelmer.com/CMSResources/Images/44-74379ATL_TableOfPolyatomicInterferences.pdf

[g] sample standard bracketing

Table S2: List of certified reference materials (CRMs). The CRMs have certified concentrations of various elements so can be used as quality control (QC) for analyte yields for plant and soil analyses. This table lists the studies that have also reported isotope compositions for the materials, as additional QC for isotope analysis and for interlaboratory comparisons.

Material	Type	Element	Reference
BCR 281	Rye grass	Si, Fe, Zn	**Frick et al. 2020, Arnold et al. 2010, 2015, Caldelas et al. 2011, Caldelas et al. 2020**, Couder et al. 2015
BCR 279	Sea Lettuce	Mg	**Bolou-Bi et al. 2009**
BCR 381	Rye flour	Mg	**Bolou-Bi et al. 2009**
BCR 383	Green beans	Mg	**Le Goff et al. 2021**
BCR 482	Lichen	Mg, Cu, Ni, Zn, Cd	**Bolou-Bi et al. 2009,** Sillerova et al. 2017, **Caldelas et al. 2011, Deng et al. 2014, Cloquet et al. 2006, Borovička et al. 2021**
BCR 62	Olive leaves	Zn	Caldelas et al. 2011, Tang et al. 2016
BCR 679	White cabbage	B, Cd	**Rosner et al. 2011, Geilert et al. 2015, Geilert et al. 2019, Moore et al. 2020**
IGGE GSB-4	Soybean	Ni	**Li et al. 2020**
IGGE GSB-12	Bean	Ni	**Li et al. 2020**
INCT Tl-1	Tea leaves	Tl	**Vanek et al. 2019**
INRA V464	Oak leaves	Ni	**Zelano et al. 2020**
IPE 126	Maize	B	**Geilert et al. 2015, Geilert et al. 2019**
SRM 1515	Apple leaves	Mg, Fe, Ni	**Shalev et al. 2018**, Garnier et al. 2017, Ratié et al. 2019
SRM 1547	Peach leaves	B, Cu, Mo	**Geilert et al. 2015, Geilert et al. 2019**, **Rosner et al. 2011**, Malinovsky & Kashulin 2018
SRM 1567b	Wheat flour	Zn, Cd	**van der Ent et al. 2021, Wiggenhauser et al. 2016, Imseng et al. 2018, 2019, Borovička et al. 2021**
SRM 1570a	Spinach leaves	Ni, Cd	Ratié et al. 2019, **Moore et al. 2020, Borovička et al. 2021**
SRM 1573a	Tomato leaves	Cu, Zn, Cd	**Ryan et al. 2013, van der Ent et al. 2021, Borovička et al. 2021**
SRM 1575a	Pine needles	Fe, Cd	Wu et al. 2021, **Borovička et al. 2021**
SRM 2384	Baking Chocolate	Cd	**Barraza et al. 2019**
SRM 8433	Corn Bran	B	**Geilert et al. 2015, Geilert et al. 2019**, **Rosner et al. 2011**
BCR 60	Light sandy soil	Zn	Caldelas et al. 2011, Caldelas et al. 2020
IGGE GSS1-7	Soils	Ni, Tl	**Li et al. 2020, Kersten et a. 2014**
SRM 2709a	San Joaquin soil	Zn, Fe, Cu, Cd	**Wiggenhauser et al. 2018, van der Ent et al. 2021**, Garnier et al. 2017, **Blotevogel et al. 2019, Barraza et al. 2019**
SRM 2710	Montana soil I	Cd	**Zhou et al. 2020, Zhong et al. 2021, 2022**
SRM 2710a	Montana soil I	Cu, Ni, Cd	Sillerova et al. 2017**, Zhong et al. 2021, 2022**
SRM 2711	Montana soil II	Cd	**Liu et al. 2020, Zhou et al. 2020, Borovička et al. 2021, Zhong et al. 2021, 2022**
SRM 2711a	Montana soil II	Cd	**Zhong et al. 2021, 2022**
TILL 11	Forest soil	Mg	**Bolou-Bi et al. 2009**

Bold references used the material for QC of isotope analysis and report isotope compositions, while the others used for yield quantification only.

Table S3: Summary of linkages between physiological and physico-chemical processes as well as knowledge gaps and corresponding research priorities. Colors: Blue = enrichment of light isotopes, red = enrichment of heavy isotopes, orange = both/unknown. Font: robust experimental evidence provided (regular), well substantiated hypotheses (italic). Empty cell = research gap.

	**root uptake**	**short distance transport (membrane transport)**	**long distance transport (xylem/phloem)**	**metabolic functionalization/storage and compartmentalization**	**others**	**proposed research priorities**
**B**					distinct B supply, cultivar, and species seems to change B isotope fractionation	- determination of B isotope fractionation during plant uptake by i) providing full B mass balances and ii) using B model plants (e.g. arabidopsis) to e.g. employ genetic approaches<br>- experimental determination of the isotope fractionation factor for cell wall binding of B by separating soluble and non-soluble B fractions from fresh plants (see example Sun et al. 2018 for dried plant material)<br>- determination of isotope fractionation for deficient and adequate B supply in model plants
		equilibrium fractionation between soluble boric acid (heavy) and borate (light)		structural binding of borate enriches cell walls in light isotopes		
**Mg**	cell wall sorption		phloem transport induces enrichment of heavy Mg in reproductive organs	binding of Mg to chlorophyll	low Mg supply changes fractionation during Mg uptake and re-translocation	- identification of the role of distinct membrane proteins on isotope fractionation during Mg uptake by employing genetic approaches with yeast and/or model plants (e.g. arabidopsis)<br>- determination of the role of vacuolar storage of Mg on the isotope fractionation between root and shoot<br>- experimental determination of equilibrium isotope fractionation of Mg binding to molecules other than chlorophyll (e.g. ATP).<br>- identifying the role of sink-source relationships by integrating isotope fractionation factors for cross-membrane transport and binding of elements to organic ligands
	binding of Mg to membrane transporter at low supply	*binding of Mg to membrane transporter at low supply*	*degradation of Mg containing molecules and subsequent re-translocation of Mg*			
**Si**	diffusion through membrane, with and without Si membrane protein	diffusion through membrane, with and without Si membrane protein	enrichment of heavy Si in tissues along the transpiration stream through precipitation of light Si into phytoliths	precipitation of Si into phytoliths	Si isotope fractionate within leaves	- determination of the role of membrane transport and Si precipitation on isotope fractionation between root and shoot.<br>- determination of the role of Lsi transporters on isotope fractionation using genetic approaches (as exemplified in Köster et al. 2009) by including biological replicates.
		precipitation of light Si into phytoliths in roots	*membrane transport in the shoot (LSi6)*			
**K**						- the method to measure K isotope ratios in plants is developed. Next steps could be to investigate the role of low and high K supply on K isotope fractionation in plants
	ion channel through distinct ionic radii and dehydration of K	*diffusion does not play ra ole during K membrane transport*	*binding of heavy isotopes to pectate in roots and leaves*			
**Ca**	root cell wall sorption			structural binding of light Ca to pectins	distinct isotope fractionation within the shoot with distinct Ca supply	- determination of isotope fraction during Ca uptake by distinct root membrane transporters that dominate at low and high Ca supply<br>- measuring Ca isotopes in storage forms (e.g. by extractions, Schmitt et al. 2018) at different Ca supply and/or in distinct phloem sources<br>- experimental determination of equilibrium and kinetic isotope fractionation of Ca binding to oxalate
	dehydration prior to membrane transport	*dehydration prior to membrane transport*	*heavy Ca in the xylem due to binding of light Ca to pectate in cell walls*	*binding of Ca to oxalate in roots and leaves*		
**Fe**	reduction prior to membrane transport, (re-)precipitation of light Fe in the apoplast, binding of Fe to phytosiderophores				redox conditions with strong impact on Fe isotopes fractionation in the soil-plant interface	- detailed investigation on Fe membrane transport by employing single cell organisms, model plants (e.g. arabidopsis, rice) as well as genetic approaches<br>- investigation on the effect of distinct Cu supply on Fe isotope fractionation during uptake<br>- determination of the impact on distinct Fe supply on Fe isotope fractionation during Fe uptake and root-to-shoot translocation<br>- focus on Fe re-translocation during grain filling periods by e.g. combined isotope and speciation measurements in the shoots.
			re-translocation of Fe(III) from leaves requires reduction to Fe(II)	*storage of Fe e.g.. leaves as Fe(III) in proteins*		

	root uptake	short distance transport (membrane transport)	long distance transport (xylem/phloem)	metabolic functionalization/storage and compartmentalization	others	proposed research priorities
**Ni**				binding of Ni to organic acids induces minor isotope fractionation	reduced isotope fractionation during Ni plant uptake with high Zn supply	- root extraction to distinguish between apoplastic and symplastic Ni in roots<br>- the role of distinct conditions in the Ni source such as pH, Fe supply, and distinct Ni speciation on Ni isotope fractionation during uptake<br>- identify processes that control the root-to-shoot transport of Ni and a systematic investigation of phloem sink-source relations during reproductive growth stages. Potentially by combining with Ni speciation measurements.
	speciation in nutrient solution, diffusion t favors light Ni	*high Ni uptake rates favor light Ni*	*Ni re-translocation during tree aging reduces Ni fractionation within shoots*			
**Cu**	reduction prior to membrane transport, Cu speciation in solution	membrane transport with and without reduction prior to transport			Cu supply, has an effect on Cu isotope fractionation during Cu uptake	- investigation of fractionation factors associated with different uptake pathways at low and high supply<br>- continuation of the yeast work of Cadiou et al. (2017) by employing similar genetic approaches in model plants<br>- systematic investigation of the role of Cu stress on isotope fractionation between roots and shoots<br>- determination of the role of transpiration during vegetative growth and re-translocation of Cu during reproductive growth phases<br>- systematic comparison of Cu isotope composition in apopoalstic and symplastic root pools
	binding during membrane transport, sorption in the apoplast		*Successive fractionation with leaf height (transpiration stream driven)*	*binding of Cu to thiols for detoxification*		
**Zn**	diffusion, speciation in solution, binding to phytosiderophores and other ligands with O and/or N donors, sorption in the apoplast	high affinity (low uptake rates) transport induces a shift towards heavy isotopes	phloem sinks enriched in light isotopes	Zn storage in root vacuoles to organic acids at high Zn concentrations	extent of isotope fractionation during Zn acquisition depends on Zn supply and uptake rates	- determination of the the role of pyhtosiderophores on Zn isotope fractionation by employing genetic approaches<br>- apply genetic appraoches to identify transporters and corresponding isotope fractionation at low and high supply<br>- systematically disentangling the role of kinetic vs. equilibrium fractionation during Zn uptake in plants<br>- focus on sink-source relationships during grain filling by combining isotope analyses with genetic and speciation approaches
	stripping off the hydration shell	*binding of Zn to membrane transporter, diffusion prior to membrane transport, binding to phytosiderophores prior to membrane transport*	*phloem transport of light isotopes due to binding and retention of heavy Zn to O and N containing ligands in senescent leaves*			
**Mo**						- the methodology to measure Mo isotopes in plants is set up. Future studies should systematically explore the impact of environmental factors, membrane proteins, and organic ligands on Mo isotope fractionation in plants
**Cd**	root uptake by Nramp5 *(binding of Cd to the transporter)*, adsorption to root surface, binding to humic acids enriches ionic Cd in heavy isotopes		xylem to phloem transfer in the nodes by OsHMA2, OsLCT1	sequestration of Cd into vacuoles causes a transport of heavy Cd from root to shoot	distinct isotope fractionation patterns between cereals, cacao, and Cd accumulators	- determination of the role of distinct environmental conditions (e.g., high and low pH) on Cd speciation in the source and Cd uptake<br>- disentangling the interplay of distinct uptake transporters on Cd isotope fractionation during plant uptake (e.g., NRAMP5, NRAMP1, IRT-1)<br>- experimental determination of the Cd isotope effect of diffusion and Cd binding to distinct organic ligands<br>- disentangling the role of Cd speciation and membrane transport on isotope fractionation<br>- determination of the role of xylem vs. phloem transport on isotope fractionation in cacao
	diffusion prior to uptake favors light isotopes	*dehydration of Cd prior to transport, binding to S donors of the membrane protein*	vacuolar sequestration of light Cd enriches phloem sinks in heavy isotopes	*vacuolar sequestration may be driven by tonoplast transport (HMA3) and chelation by thiols such as phytochelatin*		
**Tl**					Tl strongly fractionated within the plant	- determination of the role of distinct K supply on Tl isotope fractionation during Tl uptake and transport within the plant by including e.g. mutant plants with dysfunctional K uptake membrane transporters<br>- determination of equilibrium isotope fractionation for Tl binding to distinct organic ligands
	potential involvement of membrane proteins of K			*mainly bound to O ligands, S ligands play a minor role*		

References

Archer, C., Andersen, M. B., Cloquet, C., Conway, T. M., Dong, S., Ellwood, M., et al. (2017). Inter-calibration of a proposed new primary reference standard AA-ETH Zn for zinc isotopic analysis. *J. Anal. At. Spectrom.* 32, 415–419. doi:10.1039/C6JA00282J.

Arnold, T., Markovic, T., Kirk, G. J. D., Schönbächler, M., Rehkämper, M., Zhao, F. J., et al. (2015). Iron and zinc isotope fractionation during uptake and translocation in rice (*Oryza sativa*) grown in oxic and anoxic soils. *Comptes Rendus Geoscience* 347, 397–404. doi:10.1016/J.CRTE.2015.05.005.

Barraza, F., Moore, R. E. T., Rehkämper, M., Schreck, E., Lefeuvre, G., Kreissig, K., et al. (2019). Cadmium isotope fractionation in the soil-cacao systems of Ecuador: A pilot field study. *RSC Advances* 9, 34011–34022. doi:10.1039/c9ra05516a.

Baker, R. G. A., Rehkämper, M., Hinkley, T. K., Nielsen, S. G., and Toutain, J. P. (2009). Investigation of thallium fluxes from subaerial volcanism—Implications for the present and past mass balance of thallium in the oceans. *Geochimica et Cosmochimica Acta* 73, 6340–6359. doi:10.1016/j.gca.2009.07.014.

Baxter, D. C., Rodushkin, I., Engström, E., and Malinovsky, D. (2006). Revised exponential model for mass bias correction using an internal standard for isotope abundance ratio measurements by multi-collector inductively coupled plasma mass spectrometry. *Journal of Analytical Atomic Spectrometry* 21, 427. doi:10.1039/b517457k.

Blotevogel, S., Oliva, P., Sobanska, S., Viers, J., Vezin, H., Audry, S., et al. (2018). The fate of Cu pesticides in vineyard soils: A case study using δ65Cu isotope ratios and EPR analysis. *Chemical Geology* 477, 35–46. doi:10.1016/j.chemgeo.2017.11.032.

Blotevogel, S., Schreck, E., Audry, S., Saldi, G. D., Viers, J., Courjault-Radé, P., et al. (2019). Contribution of soil elemental contents and Cu and Sr isotope ratios to the understanding of pedogenetic processes and mechanisms involved in the soil-to-grape transfer (Soave vineyard, Italy). *Geoderma* 343, 72–85. doi:10.1016/j.geoderma.2019.02.015.

Bolou-Bi, E. B., Vigier, N., Brenot, A., and Poszwa, A. (2009). Magnesium isotope compositions of natural reference materials. *Geostandards and Geoanalytical Research* 33, 95–109. doi:10.1111/j.1751-908X.2009.00884.x.

Borovička, J., Ackerman, L., and Rejšek, J. (2021). Cadmium isotopic composition of biogenic certified reference materials determined by thermal ionization mass spectrometry with double spike correction. *Talanta* 221, 121389. doi:10.1016/j.talanta.2020.121389.

Borrok, D., Wanty, R., Ridley, W., Wolf, R., Lamothe, P., and Adams, M. (2007). Separation of copper, iron, and zinc from complex aqueous solutions for isotopic measurement. *Chemical Geology* 242, 400–414. doi:10.1016/j.chemgeo.2007.04.004.

Bullen, T., and Chadwick, O. (2016). Ca, Sr and Ba stable isotopes reveal the fate of soil nutrients along a tropical climosequence in Hawaii. *Chemical Geology* 422, 25–45. doi:10.1016/j.chemgeo.2015.12.008.

Caldelas, C., Dong, S., Araus, J. L., and Weiss, D. J. (2011). Zinc isotopic fractionation in Phragmites australis in response to toxic levels of zinc. *Journal of Experimental Botany* 62, 2169–2178. doi:10.1093/jxb/erq414.

Caldelas, C., Poitrasson, F., Viers, J., and Araus, J. L. (2020). Stable Zn isotopes reveal the uptake and toxicity of zinc oxide engineered nanomaterials in: Phragmites australis. *Environmental Science: Nano* 7, 1927–1941. doi:10.1039/d0en00110d.

Cameron, V., Vance, D., Archer, C., and House, C. H. (2009). A biomarker based on the stable isotopes of nickel. *Proceedings of the National Academy of Sciences* 106, 10944–10948. doi:10.1073/pnas.0900726106.

Chen, H., Tian, Z., Tuller-Ross, B., Korotev, R. L., and Wang, K. (2019). High-precision potassium isotopic analysis by MC-ICP-MS: an inter-laboratory comparison and refined K atomic weight. *J. Anal. At. Spectrom.* 34, 160–171. doi:10.1039/C8JA00303C.

Christensen, J. N., Qin, L., Brown, S. T., and Depaolo, D. J. (2018). Potassium and Calcium Isotopic Fractionation by Plants (Soybean [Glycine max], Rice [Oryza sativa], and Wheat [Triticum aestivum]). *ACS Earth and Space Chemistry* 2, 745–752. doi:10.1021/acsearthspacechem.8b00035.

Cloquet, C., Carignan, J., Libourel, G., Sterckeman, T., and Perdrix, E. (2006). Tracing Source Pollution in Soils Using Cadmium and Lead Isotopes. 6.

Couder, E., Mattielli, N., Drouet, T., Smolders, E., Delvaux, B., Iserentant, A., et al. (2015). Transpiration flow controls Zn transport in Brassica napus and Lolium multiflorum under toxic levels as evidenced from isotopic fractionation. *Comptes Rendus Geoscience* 347, 386–396. doi:10.1016/j.crte.2015.05.004.

Deng, T. H. B., Cloquet, C., Tang, Y. T., Sterckeman, T., Echevarria, G., Estrade, N., et al. (2014). Nickel and zinc isotope fractionation in hyperaccumulating and nonaccumulating plants. *Environmental Science and Technology* 48, 11926–11933. doi:10.1021/es5020955.

Frick, D. A., Remus, R., Sommer, M., Augustin, J., and von Blanckenburg, F. (2020). Silicon isotope fractionation and uptake dynamics of three crop plants: laboratory studies with transient silicon concentrations. Biogeochemistry: Stable Isotopes & Other Tracers doi:10.5194/bg-2020-66.

Garnier, J., Garnier, J. M., Vieira, C. L., Akerman, A., Chmeleff, J., Ruiz, R. I., et al. (2017). Iron isotope fingerprints of redox and biogeochemical cycling in the soil-water-rice plant system of a paddy field. *Science of the Total Environment* 574, 1622–1632. doi:10.1016/j.scitotenv.2016.08.202.

Geilert, S., Vogl, J., and Rosner, M. (2015). Boron Isotope Fractionation in Bell Pepper. *Mass Spectrometry & Purification Techniques* 01, 1–6. doi:10.4172/mso.1000101.

Geilert, S., Vogl, J., Rosner, M., and Eichert, T. (2019). Boron isotope variability related to boron speciation (change during uptake and transport) in bell pepper plants and SI traceable n(11B)/n(10B) ratios for plant reference materials. *Rapid Communications in Mass Spectrometry* 33, 1137–1147. doi:10.1002/rcm.8455.

Georg, R. B., Reynolds, B. C., Frank, M., and Halliday, A. N. (2006). New sample preparation techniques for the determination of Si isotopic compositions using MC-ICPMS. *Chemical Geology* 235, 95–104. doi:10.1016/j.chemgeo.2006.06.006.

Imseng, M., Wiggenhauser, M., Keller, A., Müller, M., Rehkämper, M., Murphy, K., et al. (2018). Fate of Cd in Agricultural Soils: A Stable Isotope Approach to Anthropogenic Impact, Soil Formation, and Soil-Plant Cycling. *Environmental Science & Technology* 52, 1919–1928. doi:10.1021/acs.est.7b05439.

Kerl, C. F., Lohmayer, R., Bura-Nakić, E., Vance, D., and Planer-Friedrich, B. (2017). Experimental Confirmation of Isotope Fractionation in Thiomolybdates Using Ion Chromatographic Separation and Detection by Multicollector ICPMS. *Analytical Chemistry* 89, 3123–3129. doi:10.1021/acs.analchem.6b04898.

Kersten, M., Xiao, T., Kreissig, K., Brett, A., Coles, B. J., and Rehkämper, M. (2014). Tracing anthropogenic thallium in soil using stable isotope compositions. *Environmental Science and Technology* 48, 9030–9036. doi:10.1021/es501968d.

Kidder, J. A., Voinot, A., Sullivan, K. V., Chipley, D., Valentino, M., Layton-Matthews, D., et al. (2020). Improved ion-exchange column chromatography for Cu purification from high-Na matrices and isotopic analysis by MC-ICPMS. *Journal of Analytical Atomic Spectrometry* 35, 776–783. doi:10.1039/c9ja00359b.

Köster, J. R., Bol, R., Leng, M. J., Parker, A. G., Sloane, H. J., and Ma, J. F. (2009). Effects of active silicon uptake by rice on 29 Si fractionation in various plant parts. Rapid Commun. Mass Spectrom. 23, 2398–2402. doi:10.1002/rcm.3971.

Le Goff, S., Albalat, E., Dosseto, A., Godin, J., and Balter, V. (2021). Determination of magnesium isotopic ratios of biological reference materials via multi-collector inductively coupled plasma mass spectrometry. *Rapid Commun Mass Spectrom* 35. doi:10.1002/rcm.9074.

Li, W., Beard, B. L., and Li, S. (2016). Precise measurement of stable potassium isotope ratios using a single focusing collision cell multi-collector ICP-MS. *Journal of Analytical Atomic Spectrometry* 31, 1023–1029. doi:10.1039/c5ja00487j.

Li, W., Zhu, J. M., Tan, D., Han, G., Zhao, Z., and Wu, G. (2020). The δ60/58Ni Values of Twenty-Six Selected Geological Reference Materials. *Geostandards and Geoanalytical Research* 44, 523–535. doi:10.1111/ggr.12321.

Liu, A., Zhou, Z., Yi, Y., and Chen, G. (2020). Transcriptome analysis reveals the roles of stem nodes in cadmium transport to rice grain. *BMC Genomics* 21, 127. doi:10.1186/s12864-020-6474-7.

Malinovsky, D., and Kashulin, N. A. (2018). Molybdenum isotope fractionation in plants measured by MC-ICPMS. *Analytical Methods* 10, 131–137. doi:10.1039/c7ay02316b.

Marković, T., Manzoor, S., Humphreys-Williams, E., Kirk, G. J. D., Vilar, R., and Weiss, D. J. (2017). Experimental Determination of Zinc Isotope Fractionation in Complexes with the Phytosiderophore 2′-Deoxymugeneic Acid (DMA) and Its Structural Analogues, and Implications for Plant Uptake Mechanisms. *Environmental Science and Technology* 51, 98–107. doi:10.1021/acs.est.6b00566.

Moeller, K., Schoenberg, R., Pedersen, R.-B., Weiss, D., and Dong, S. (2012). Calibration of the New Certified Reference Materials ERM-AE633 and ERM-AE647 for Copper and IRMM-3702 for Zinc Isotope Amount Ratio Determinations. *Geostandards and Geoanalytical Research* 36, 177–199. doi:10.1111/j.1751-908X.2011.00153.x.

Moore, R. E. T., Larner, F., Coles, B. J., and Rehkämper, M. (2017). High Precision Zinc Stable Isotope Measurement of Certified Biological Reference Materials Using the Double Spike Technique and Multiple Collector-ICP-MS. *Analytical and Bioanalytical Chemistry* 409, 2941–2950. doi:10.1007/s00216-017-0240-y.

Moore, R. E. T., Ullah, I., de Oliveira, V. H., Hammond, S. J., Strekopytov, S., Tibbett, M., et al. (2020). Cadmium isotope fractionation reveals genetic variation in Cd uptake and translocation by *Theobroma cacao* and role of natural resistance-associated macrophage protein 5 and heavy metal ATPase-family transporters. *Horticulture Research* 7, 71. doi:10.1038/s41438-020-0292-6.

Morgan, L. E., Santiago Ramos, D. P., Davidheiser-Kroll, B., Faithfull, J., Lloyd, N. S., Ellam, R. M., et al. (2018). High-precision 41K/39K measurements by MC-ICP-MS indicate terrestrial variability of: δ 41K. *Journal of Analytical Atomic Spectrometry* 33, 175–186. doi:10.1039/c7ja00257b.

Murphy, K., Rehkämper, M., Kreissig, K., Coles, B., and van de Flierdt, T. (2016). Improvements in Cd stable isotope analysis achieved through use of liquid–liquid extraction to remove organic residues from Cd separates obtained by extraction chromatography. *Journal of Analytical Atomic Spectrometry* 31, 319–327. doi:10.1039/C5JA00115C.

Oelze, M., Schuessler, J. A., and von Blanckenburg, F. (2016). Mass bias stabilization by Mg doping for Si stable isotope analysis by MC-ICP-MS. *J. Anal. At. Spectrom.* 31, 2094–2100. doi:10.1039/C6JA00218H.

Opfergelt, S., Georg, R. B., Delvaux, B., Cabidoche, Y. M., Burton, K. W., and Halliday, A. N. (2012). Mechanisms of magnesium isotope fractionation in volcanic soil weathering sequences, Guadeloupe. *Earth and Planetary Science Letters* 341–344, 176–185. doi:10.1016/j.epsl.2012.06.010.

Quitte, G., and Oberli, F. (2006). Quantitative extraction and high precision isotope measurements of nickel by MC-ICPMS. *J. Anal. At. Spectrom.* 21, 1249. doi:10.1039/b607569j.

Rader, S. T., Maier, R. M., Barton, M. D., and Mazdab, F. K. (2019). Uptake and Fractionation of Thallium by Brassica juncea in a Geogenic Thallium-Amended Substrate. doi:10.1021/acs.est.8b06222.

Ratié, G., Quantin, C., Maia De Freitas, A., Echevarria, G., Ponzevera, E., and Garnier, J. (2019). The behavior of nickel isotopes at the biogeochemical interface between ultramafic soils and Ni accumulator species. *Journal of Geochemical Exploration*. doi:10.1016/j.gexplo.2018.10.008.

Rosner, M., Pritzkow, W., Vogl, J., and Voerkelius, S. (2011). Development and validation of a method to determine the boron isotopic composition of crop plants. *Analytical Chemistry* 83, 2562–2568. doi:10.1021/ac102836h.

Ryan, B. M., Kirby, J. K., Degryse, F., Harris, H., McLaughlin, M. J., and Scheiderich, K. (2013). Copper speciation and isotopic fractionation in plants: uptake and translocation mechanisms. *New Phytologist* 199, 367–378. doi:10.1111/nph.12276.

Schmitt, A.-D., Gangloff, S., Cobert, F., Lemarchand, D., Stille, P., and Chabaux, F. (2009). High performance automated ion chromatography separation for Ca isotope measurements in geological and biological samples. *J. Anal. At. Spectrom.* 24, 1089. doi:10.1039/b903303c.

Schmitt, A. D., Borrelli, N., Ertlen, D., Gangloff, S., Chabaux, F., and Osterrieth, M. (2018). Stable calcium isotope speciation and calcium oxalate production within beech tree (Fagus sylvatica L.) organs. Biogeochemistry 137, 197–217. doi:10.1007/s10533-017-0411-0.

Schoenberg, R., and von Blanckenburg, F. (2005). An assessment of the accuracy of stable Fe isotope ratio measurements on samples with organic and inorganic matrices by high-resolution multicollector ICP-MS. *International Journal of Mass Spectrometry* 242, 257–272. doi:10.1016/j.ijms.2004.11.025.

Šillerová, H., Chrastný, V., Vítková, M., Francová, A., Jehlička, J., Gutsch, M. R., et al. (2017). Stable isotope tracing of Ni and Cu pollution in North-East Norway: Potentials and drawbacks. *Environmental Pollution* 228, 149–157. doi:10.1016/j.envpol.2017.05.030.

Shalev, N., Farkaš, J., Fietzke, J., Novák, M., Schuessler, J. A., Pogge von Strandmann, P. A. E., et al. (2018). Mg Isotope Interlaboratory Comparison of Reference Materials from Earth-Surface Low-Temperature Environments. *Geostand Geoanal Res* 42, 205–221. doi:10.1111/ggr.12208.

Strelow, F. (1980). Improved separation of iron from copper and other elements by anion-exchange chromatography on a 4% cross-linked resin with high concentrations of hydrochloric acid. *Talanta* 27, 727–732. doi:10.1016/0039-9140(80)80166-4.

Sun, A., Xu, Q., Wei, G., Zhu, H., and Chen, X. (2018). Differentiation analysis of boron isotopic fractionation in different forms within plant organ samples. Phytochemistry 147, 9–13. doi:10.1016/j.phytochem.2017.12.012.

Tang, Y.-T., Cloquet, C., Deng, T.-H.-B., Sterckeman, T., Echevarria, G., Yang, W.-J., et al. (2016). Zinc Isotope Fractionation in the Hyperaccumulator *Noccaea caerulescens* and the Nonaccumulating Plant *Thlaspi arvense* at Low and High Zn Supply. *Environmental Science & Technology* 50, 8020–8027. doi:10.1021/acs.est.6b00167.

Vaněk, A., Holubík, O., Oborná, V., Mihaljevič, M., Trubač, J., Ettler, V., et al. (2019). Thallium stable isotope fractionation in white mustard: Implications for metal transfers and incorporation in plants. *Journal of Hazardous Materials* 369, 521–527. doi:10.1016/j.jhazmat.2019.02.060.

van der Ent, A., Nkrumah, P. N., Aarts, M. G. M., Baker, A. J. M., Degryse, F., Wawryk, C., et al. (2021). Isotopic signatures reveal zinc cycling in the natural habitat of hyperaccumulator Dichapetalum gelonioides subspecies from Malaysian Borneo. *BMC Plant Biol* 21, 437. doi:10.1186/s12870-021-03190-4.

von Blanckenburg, F., Oelze, M., Schmid, D. G., van Zuilen, K., Gschwind, H.-P., Slade, A. J., et al. (2014). An iron stable isotope comparison between human erythrocytes and plasma. *Metallomics* 6, 2052–2061. doi:10.1039/C4MT00124A.

Wang, Y., Wu, B., Berns, A. E., Xing, Y., Kuhn, A. J., and Amelung, W. (2020). Magnesium isotope fractionation reflects plant response to magnesium deficiency in magnesium uptake and allocation: a greenhouse study with wheat. *Plant and Soil* 455, 93–105. doi:10.1007/s11104-020-04604-2.

Wei, R., Guo, Q., Wen, H., Yang, J., Peters, M., Zhu, C., et al. (2015). An analytical method for precise determination of the cadmium isotopic composition in plant samples using multiple collector inductively coupled plasma mass spectrometry. *Analytical Methods* 7, 2479–2487. doi:10.1039/c4ay02435d.

Wiederhold, J. G., Kraemer, S. M., Teutsch, N., Borer, P. M., Halliday, A. N., and Kretzschmar, R. (2006). Iron isotope fractionation during proton-promoted, ligand-controlled, and reductive dissolution of goethite. *Environmental Science and Technology* 40, 3787–3793. doi:10.1021/es052228y.

Wiggenhauser, M., Bigalke, M., Imseng, M., Keller, A., Archer, C., Wilcke, W., et al. (2018). Zinc isotope fractionation during grain filling of wheat and a comparison of zinc and cadmium isotope ratios in identical soil-plant systems. *New Phytologist* 219, 195–205. doi:10.1111/nph.15146.

Wiggenhauser, M., Bigalke, M., Imseng, M., Müller, M., Keller, A., Murphy, K., et al. (2016). Cadmium isotope fractionation in soil–wheat systems. *Environmental Science & Technology* 50, 9223–9231. doi:10.1021/acs.est.6b01568.

Wu, B., Wang, Y., Berns, A. E., Schweitzer, K., Bauke, S. L., Bol, R., et al. (2021). Iron isotope fractionation in soil and graminaceous crops after 100 years of liming in the long-term agricultural experimental site at Berlin-Dahlem, Germany. *Eur J Soil Sci* 72, 289–299. doi:10.1111/ejss.12944.

Zelano, I. O., Cloquet, C., van der Ent, A., Echevarria, G., Gley, R., Landrot, G., et al. (2020). Coupling nickel chemical speciation and isotope ratios to decipher nickel dynamics in the Rinorea cf. bengalensis-soil system in Malaysian Borneo. *Plant and Soil* 454, 225–243. doi:10.1007/s11104-020-04541-0.

Zhong, S., Li, X., Li, F., Liu, T., Huang, F., Yin, H., et al. (2021). Water Management Alters Cadmium Isotope Fractionation between Shoots and Nodes/Leaves in a Soil-Rice System. *Environmental Science & Technology*, acs.est.0c04713. doi:10.1021/acs.est.0c04713.

Zhong, S., Li, X., Li, F., Huang, Y., Liu, T., Yin, H., et al. (2022). Cadmium uptake and transport processes in rice revealed by stable isotope fractionation and Cd-related gene expression. *Science of The Total Environment* 806, 150633. doi:10.1016/j.scitotenv.2021.150633.

Zhou, J., Li, Z., Liu, M., Yu, H., Wu, L., Huang, F., et al. (2020). Cadmium isotopic fractionation in the soil − plant system during repeated phytoextraction with a cadmium hyperaccumulating plant species. *Environmental Science & Technology* 54, 13598–13609. doi:10.1021/acs.est.0c03142.
